# Identification of protein biomarkers for prediction of response to platinum‐based treatment regimens in patients with non‐small cell lung cancer

**DOI:** 10.1002/1878-0261.13555

**Published:** 2024-01-25

**Authors:** Franziska Böttger, Teodora Radonic, Idris Bahce, Kim Monkhorst, Sander R. Piersma, Thang V. Pham, Anne‐Marie C. Dingemans, Lisa M. Hillen, Mariacarmela Santarpia, Elisa Giovannetti, Egbert F. Smit, Sjaak A. Burgers, Connie R. Jimenez

**Affiliations:** ^1^ Department of Medical Oncology, Cancer Center Amsterdam Amsterdam UMC – location VUmc The Netherlands; ^2^ OncoProteomics Laboratory, Cancer Center Amsterdam Amsterdam UMC – location VUmc The Netherlands; ^3^ Department of Pathology Amsterdam UMC – location VUmc The Netherlands; ^4^ Department of Pulmonary Diseases Amsterdam UMC – location VUmc The Netherlands; ^5^ Division of Pathology The Netherlands Cancer Institute – Antoni van Leeuwenhoek Hospital Amsterdam The Netherlands; ^6^ Department of Pulmonary Diseases, GROW School for Oncology & Developmental Biology Maastricht University Medical Center The Netherlands; ^7^ Department of Pulmonary Diseases Erasmus Medical Centre Rotterdam The Netherlands; ^8^ Department of Pathology Maastricht University Medical Center The Netherlands; ^9^ Medical Oncology Unit, Department of Human Pathology “G. Barresi” University of Messina Italy; ^10^ Cancer Pharmacology Lab Fondazione Pisana per la Scienza Pisa Italy; ^11^ Division of Thoracic Oncology The Netherlands Cancer Institute – Antoni van Leeuwenhoek Hospital Amsterdam The Netherlands; ^12^ Department of Pulmonary Diseases Leiden University Medical Center The Netherlands

**Keywords:** adjuvant chemotherapy, biomarker, lung cancer, mass‐spectrometry, platinum, proteomics

## Abstract

The majority of patients with resected stage II‐IIIA non‐small cell lung cancer (NSCLC) are treated with platinum‐based adjuvant chemotherapy (ACT) in a one‐size‐fits‐all approach. However, a significant number of patients do not derive clinical benefit, and no predictive patient selection biomarker is currently available. Using mass spectrometry‐based proteomics, we have profiled tumour resection material of 2 independent, multi‐centre cohorts of in total 67 patients with NSCLC who underwent ACT. Unsupervised cluster analysis of both cohorts revealed a poor response/survival sub‐cluster composed of ~ 25% of the patients, that displayed a strong epithelial‐mesenchymal transition signature and stromal phenotype. Beyond this stromal sub‐population, we identified and validated platinum response prediction biomarker candidates involved in pathways relevant to the mechanism of action of platinum drugs, such as DNA damage repair, as well as less anticipated processes such as those related to the regulation of actin cytoskeleton. Integration with pre‐clinical proteomics data supported a role for several of these candidate proteins in platinum response prediction. Validation of one of the candidates (HMGB1) in a third independent patient cohort using immunohistochemistry highlights the potential of translating these proteomics results to clinical practice.

## Graphical abstract

Protein expression profiles of formalin‐fixed paraffin‐embedded tumour resection material of patients with early‐stage non‐small cell lung cancer who underwent platinum‐based adjuvant chemotherapy were generated using mass‐spectrometry. The subsequent identification and validation of key protein determinants of platinum response in these patients provides insights into predictive biomarkers, the landscape of platinum resistance mechanisms, and approaches to improve therapeutic efficacy.

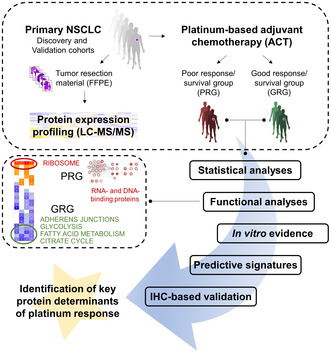

**Abbreviations**
ACT: adjuvant chemotherapy
DEPs: differentially expressed proteins
DSB: double strand break
ECM: extracellular matrix
EMT: epithelial–mesenchymal transition
FDR: false discovery rate
FFPE: formalin‐fixed paraffin‐embedded
GRG: good response/survival group
GSEA: gene set enrichment analysis
IHC: immunohistochemistry
LC–MS/MS: liquid chromatography tandem mass‐spectrometry
LUAD: lung adenocarcinoma
LUSC: lung squamous carcinoma
MMR: mismatch repair
NER: nucleotide excision repair
NES: normalized enrichment score
NSCLC: non‐small cell lung cancer
OS: overall survival
PPI: protein–protein interaction
PR/S: poor response/survival (cluster)
PRG: poor response/survival group
RFS: recurrence‐free survival
SC: spectral count
TCP: tumour cell percentage
TMA: tissue microarray
UT: untreated

## 1 Introduction

Lung cancer, of which non‐small cell lung cancer (NSCLC) is the most frequently diagnosed type, is the deadliest malignancy worldwide, accounting for 18 % of all cancer deaths in 2020 [1]. The overall 5‐year survival rate is less than 20% and has increased only marginally in the last decades [2]. This poor overall survival (OS) is mainly due to late detection of the tumour, which causes most patients to present with (inoperable or) metastasized disease.

Despite improved lung cancer screening protocols and a shift towards detecting lower‐stage cancers [3, 4], the proportion of patients with NSCLC who have early‐stage (I‐IIIA) disease, where surgery serves as the cornerstone of curative treatment, remains only one‐third. Platinum‐based adjuvant chemotherapy (ACT) regimens after surgery are standard care in stage II‐IIIA patients with NSCLC, based on a 4–5% absolute OS benefit after 5 years [5]. In addition, while immunotherapy is now entering the adjuvant therapy arena for stage IB to IIIA resectable NSCLC, cytotoxic chemotherapy remains part of the adjuvant therapeutic arsenal for this patient population [6, 7, 8]. In early and late stage NSCLC, patient selection for therapies is based on immunohistochemistry (IHC) for PD‐L1 and the presence of targetable driver mutations (EGFR, KRAS G12C etc.) or driver fusions (ALK, ROS1, RET). It is widely acknowledged that patient selection for therapies based on specific biomarkers is the way to go forward in precision oncology [9]. However, there are no predictive biomarkers in tissue for response to chemotherapy.

Cisplatin or carboplatin are the major components of most chemotherapies (in combination with drugs such as pemetrexed or gemcitabine). As alkylating agents, these platinum drugs damage DNA via the formation of cross‐links, ultimately preventing DNA replication and RNA transcription, leading to apoptotic cell death and immune responses. Nucleotide excision repair (NER) is the major DNA repair pathway in platinum‐induced lesions, and enhanced repair and tolerance of platinum‐induced DNA damage is one of the key mechanisms involved in platinum resistance. Other processes involved in determining sensitivity and resistance include regulation of drug accumulation and detoxification as well as alterations in cell (survival/death) signalling and changes in the tumour microenvironment [10]. Of note, besides DNA, cisplatin has also been shown to modify other molecules, such as RNA [11], adding yet another putative source from which platinum toxicity can originate.

Many genomic instable tumours are characterized by the loss of functionality in one or several DNA repair pathways. In this context, optimizing treatment according to tumour status for DNA repair biomarkers could predict response to DNA damaging therapies such as platinum‐based ACT and might substantially improve the response of individual patients' tumours. Therefore, several DNA repair proteins, including NER protein ERCC1, the mismatch repair (MMR) proteins MSH2 and MSH6, and double strand break (DSB) repair proteins BRCA1/2, have been explored in lung cancer for their use as prognostic and/or predictive biomarkers of response to chemotherapy [12]. A recurring observation is that low ERCC1 expression is associated with sensitivity to platinum [13], but the results published in retrospective and prospective studies are not always consistent [14]. Protein profiling by mass‐spectrometry may be advantageous in this context as it provides a read‐out of multiple proteins in a larger context of (DNA repair) pathways. For example, in our study of BRCA1‐deficient breast cancer [15], we identified over 400 proteins that were upregulated in BRCA1‐deficient tumours as compared to BRCA1‐proficient tumours. These regulated proteins were linked to 29 non‐redundant nuclear protein complexes that underscored the importance of alterations in multiple DNA repair pathways and chromatin remodelling. Based on highly connected nodes in these complexes, a BRCA1‐deficiency signature was constructed with value for selecting patients with “BRCAness” in independent cohorts. This study clearly shows the potential of unbiased proteomics in constructing biologically relevant signatures with clinical value.

Despite these efforts and the reported putative association of > 900 genes/proteins with platinum resistance in various cell line, xenograft and human cancer tissue studies [10], no patient selection biomarker is currently available for platinum treatment. The availability of biomarkers (detected in tissue or body fluids) that can predict a patient's response to platinum‐based therapies could help prevent unnecessary treatment and the associated toxicities in patients who are resistant to platinum drugs, and provide guidance for alternative non‐platinum‐based regimens. By performing mass spectrometry‐based proteomics in treatment‐naive tumour resection material of patients with NSCLC who underwent platinum‐based ACT, we describe known and novel biological aspects of platinum sensitivity and resistance, and discover and validate putative protein biomarkers that can predict response to platinum treatment.

## 2 Materials and methods

### 2.1 Formalin‐fixed, paraffin‐embedded (FFPE) tissue collection

Formalin‐fixed, paraffin‐embedded specimens of patients who underwent radical surgical resection for primary NSCLC between 2004 and 2014 were waived by the respective biobanks: Amsterdam UMC/VU Medical Center biobank (BUP2016‐032; VUmc patient sub‐cohort), the NKI‐AVL Core Facility Molecular Pathology & Biobanking (CFMPB) of the Netherlands Cancer Institute – Antoni van Leeuwenhoek Hospital, Amsterdam (CFMPB238; NKI patient sub‐cohort) and the Maastricht Pathology Tissue Collection (MPTC; via BUP2019‐062; MUMC patient sub‐cohort). The use of pre‐existing archived and de‐identified samples does not fall within the scope of the Medical Research Involving Human Subjects Act in the Netherlands. Collection, storage and use of tissue and patient data were performed in agreement with the “Code for Proper Secondary Use of Human Tissue in the Netherlands” developed by the Dutch Federation of Medical Scientific Societies (FMWV). The inclusion criteria were as follows: (a) all patients underwent complete resection of primary NSCLC (both lung adenocarcinoma (LUAD) and lung squamous carcinoma (LUSC) cases were included); (b) patients received platinum‐based ACT following surgery (with the exception of a small cohort of patients that were left untreated (UT) following surgery and were used as part of the validation process); (c) at least 36 months of survival follow‐up information was available; and (d) samples had a tumour cell percentage (TCP) of at least 30% (for the discovery cohort, 40% for the validation cohort), as determined by two independent pathologists. Patients who received additional peri‐operative treatment besides platinum‐based ACT (e.g. radiotherapy, neo‐ACT or participation in clinical trials) were not considered. Cancer staging was performed in accordance with the TNM classification (version 7) of the Union for International Cancer Control [16]. Recurrence‐free survival (RFS) was defined as the time between first chemotherapy (ACT sub‐cohort) or resection (UT sub‐cohort) and recurrence (local, regional or distant) or death due to any cause. Clinico‐pathological characteristics of both discovery (consisting of samples from VUmc and NKI patients) and validation (consisting of samples from VUmc and MUMC patients) cohorts are reported in Table 1, Table S1 and Fig. S1.

**Table 1:** Clinico‐pathological characteristics of discovery and validation patient cohorts analysed by proteomics.

	Discovery		Validation			
	ACT		ACT		UTc	
	*N* (45)	%	*N* (22)	%	*N* (10)	%
Age, median (mean ± SD), years	61.0 (60.3 ± 7.6)		66.5 (64.5 ± 8.0)		59.5 (62.7 ± 9.9)	
Sex						
Male	26	57.8	11	50.0	6	60.0
Female	19	42.2	11	50.0	4	40.0
Subtype						
LUAD	26	57.8	14	63.6	10	100
LUSC	19	42.2	8	36.4	0	0.0
Sub‐cohort						
VUmc	33	73.3	7	31.8	10	100
NKI	12	26.7	0	0.0	0	0.0
MUMC	0	0.0	15	68.2	0	0.0
pStage^a^						
IA	1	2.2	0	0.0	2	20.0
IB	4	8.9	2	9.1	2	20.0
IIA	19	42.2	8	36.4	3	30.0
IIB	5	11.1	4	18.2	1	10.0
IIIA	15	33.3	6	27.3	1	10.0
IIIB	0	0.0	1	4.5	0	0.0
IV	1	2.2	0	0.0	1	10.0
Unknown	0	0.0	1	4.5	0	0.0
Platinum drug						
Cisplatin	31	68.9	20	90.9		
Carboplatin	11	24.4	1	4.5		
Both, sequential	3	6.7	0	0.0		
Unknown	0	0.0	1	4.5		
Combination drug						
Gemcitabine	25	55.6	8	36.4		
Pemetrexed	16	35.6	9	40.9		
Both, sequential	0	0.0	3	13.6		
Other	4	8.9	1	4.5		
Unknown	0	0.0	1	4.5		
TCP^b^, median (mean ± SD), %	55 (54.6 ± 10.6)		45 (46.8 ± 12.2)		45 (48.0 ± 9.0)	

^a^ Cancer staging was performed in accordance with the TNM classification (version 7) of the Union for International Cancer Control [16].

^b^ TCP, tumour cell percentage, average estimations of 2 independent pathologists.

### 2.2 FFPE tissue preparation for mass‐spectrometry analysis

Formalin‐fixed, paraffin‐embedded tissue sections (6 × 10 μm thickness) were cut from paraffin blocks (with reference slides for staining with haematoxylin and eosin (HE) according to standard procedures being taken at regular intervals during cutting). Proteins were extracted and subsequently digested to peptides as described previously [17], with minor modifications. Following separation of proteins using SDS/PAGE, each sample lane was cut from the gel as a single band before being subjected to protein digestion. Peptides were extracted and desalted using Oasis HLB cartridges (Waters Chromatography B.V, Etten‐Leur, The Netherlands), and peptide concentrations were determined using Pierce Quantitative Colorimetric Peptide Assay (Thermo Scientific, Bremen, Germany). Subsequently, peptide eluates (4 μg) were lyophilized and re‐dissolved in 20 μL 4% acetonitrile + 0.5% Trifluoroacetic acid.

### 2.3 Liquid chromatography tandem mass‐spectrometry (LC–MS/MS) analysis of FFPE samples

LC–MS/MS was performed as described previously [17]. Peptides (5 μL) were separated by an Ultimate 3000 nanoLC‐MS/MS system (Thermo Fisher, Bremen, Germany), equipped with a 20 cm × 75 μm ID fused silica column custom packed with 1.9 μm 120 A° ReproSil Pur C18 aqua (Dr Maisch GMBH, Ammerbuch‐Entringen, Germany). After injection, peptides were trapped at 6 μL·min^−1^ on a 10 mm × 100 μm ID trap column packed with 5 μm 120 A° ReproSil Pur C18 aqua in 0.05% FA. Peptides were separated at 300 nL·min^−1^ in a 10–40% gradient (buffer A: 0.5% acetic acid, buffer B: 80% ACN, 0.5% acetic acid) in 60 min (90‐min inject‐to‐inject). Eluting peptides were ionized at a potential of +2 kVa into a Q Exactive mass spectrometer (Thermo Fisher, Bremen, Germany). Intact masses were measured at resolution 70 000 (at m/z 200) in the orbitrap using an AGC target value of 3E6 charges. The top 10 peptide signals (charge‐states 2+ and higher) were submitted to MS/MS in the HCD (higher‐energy collision) cell (1.6 amu isolation width, 25% normalized collision energy). MS/MS spectra were acquired at resolution 17 500 (at m/z 200) in the orbitrap using an AGC target value of 1E6 charges, a maxIT of 60 ms, and an underfill ratio of 0.1%. Dynamic exclusion was applied with a repeat count of 1 and an exclusion time of 30 s.

### 2.4 Processing and analysis of proteomics data

MS/MS spectra were searched against the Swissprot Homo sapiens reference proteome FASTA file (release January 2018, 42 259 canonical and isoform entries for the discovery cohort and release April 2020, 42 347 canonical and isoform entries for the validation cohort) using MaxQuant versions 1.6.0.16 (discovery cohort) and 1.6.10.43 (validation cohort) [18]. Search settings were selected as described previously [17]. Enzyme specificity was set to trypsin, and up to two missed cleavages were allowed. Cysteine carbamidomethylation (Cys, +57.021464 Da) was treated as fixed modification and methionine oxidation (Met, +15.994915 Da) and N‐terminal acetylation (N‐terminal, +42.010565 Da) as variable modifications. Peptide precursor ions were searched with a maximum mass deviation of 4.5 ppm and fragment ions with a maximum mass deviation of 20 ppm. Peptide and protein identifications were filtered at an false discovery rate (FDR) of 1% using the decoy database strategy. The minimal peptide length was 7 amino acids. Proteins that could not be differentiated based on MS/MS spectra alone were grouped into protein groups (default MaxQuant settings). Searches were performed with the label‐free quantification option selected. Proteins were quantified by spectral counting [19]. Raw spectral counts were normalized on the sum of spectral counts for all identified proteins in a particular sample, relative to the average sample sum determined with all samples (Tables S2 and S3). To find statistically significant differences in normalized counts between sample groups, we applied the beta‐binomial test [20], which takes into account within‐sample and between‐sample variation (Tables S4 and S5).

### 2.5 Survival analysis

Kaplan–Meier survival curves were constructed and analysed using the survminer package in r. RFS, defined as the time between first chemotherapy (ACT sub‐cohorts) or resection (UT sub‐cohort) and recurrence (local, regional or distant) or death due to any cause) was used as the metric for clinical outcome. *P* values were calculated using the log‐rank test.

### 2.6 Data analysis and visualization

Heatmaps were generated using the ComplexHeatmap package in r (version 4.0.2). Boxplots were made using the ggplot2 package in r, Microsoft Excel (version 2016) or graphpad prism (version 7.04). For gene set enrichment analysis (GSEA) using the fgsea package in r (nperm = 5000, gseaParam = 1, minSize = 15, maxSize = 500), a rank metric score based on the negative log10 *P*‐value multiplied by the sign of log2 fold‐change was used as input. Network analysis was performed using the string tool, version 11.0 [21] and visualized using cytoscape, version 3.7.2 [22], employing Cytoscape MCL cluster plugin. Functional enrichment analysis was performed using g:Profiler, version e99_eg46_p14_0f89727 [23]. ESTIMATE analysis was performed using the estimate package in r. Venn diagrams were constructed using venny v2.1.0 [24].

### 2.7 Generation of predictive signatures

To construct predictive models and assign discriminative signature scores, we performed either exhaustive search of 4‐protein combinations (557 845 combinations) or 500 000 random probing of 5‐protein combinations using logistic regression, and subsequently recorded the frequency of proteins appearing in the best performing classifiers according to leave‐one‐out cross validation. We used a stepwise logistic regression to construct a classifier, starting from either the protein with highest area under the curve (AUC) or the most robust marker (see Section 2.8).

### 2.8 Calculation of robustness and signature scores

The cumulative robustness score of a protein was based on (a) spectral count (SC):intensity correlation (< 0.75, score 0; 0.75–0.85, score 1; 0.85–0.95, score 2; > 0.95, score 3); (b) average abundance in upregulated group (≤ 5 SCs in both cohorts, score 0; < 15 in at least one cohort, score 1; ≥ 15 in both cohorts, score 2; ≥ 30 in both cohorts, score 3); (c) significance threshold (T1 in at least 1 group, score 0; T2:T2, score 1; T2:T3 or T3:T2, score 2; T3 in both cohorts, score 3; T1 = *P*‐value < 0.05, T2 = T1 + data presence ≥ 60% in upregulated group, T3 = T2 + fold‐change > 1.5/< −1.5) and (d) number of unique peptides relative to the estimated molecular weight of a protein (< 0.1 in at least one cohort, score 0; < 0.2 in at least 1 cohort, score 1; > 0.2 in both cohorts, score 2), see Table S6. The signature score was based on a proteins position in the top 30 lists of best 4‐ and 5‐ protein signature combinations (Section 2.7, Table S6; top 10, score 3; top20, score 2, top 30, score 1; not in top 30, score 0) and whether it featured in any of the 4 stepwise logistic regression signatures (see Section 2.7, Table S6; yes, score 1; no, score 0).

### 2.9 Immunohistochemistry

HMGB1 (D3E5) antibody (#6893) from Cell Signaling Technology (Bioke, Leiden, The Netherlands) was used to stain 4 μm thick FFPE tissue sections. The sections were incubated for 48 min at a dilution of 1 : 100 in Dako Antibody Diluent (Agilent, Amstelveen, The Netherlands, #S3022). Prior to staining, the tissue sections underwent retrieval in cell conditioning 1 solution (Roche, Almere, The Netherlands, #950‐124) using a Ventana Benchmark Ultra machine (Roche, Almere, The Netherlands). Detection was performed using OptiView DAB IHC detection kit (Roche, Almere, The Netherlands, 760‐700).

Cellular localization and staining intensity of HMGB1 protein was assessed by an experienced histopathologist.

### 2.10 Tissue microarray (TMA) construction and immunohistochemistry

A total of 74 Caucasian treatment‐naïve patients with NSCLC treated at Humanitas Clinical Institute (Rozzano, Milan, Italy), Livorno Civil Hospital (Livorno, Italy), University Hospital Antwerp (Antwerp, Belgium) and the Onze‐Lieve‐Vrouw‐Hospital (Aalst, Belgium), were enrolled between 2004 and 2013. The selection was based on the availability of surgically resected FFPE specimens, diagnosis of histologically confirmed NSCLC, and treatment with adjuvant platinum‐based regimens. The study on patients' specimens was approved by the local ethics committees (Ethics Committee of Istituto Clinico Humanitas (Rozzano, Milan, Italy) – Ref‐No. 165/17, Comitato Etico di Area Vasta Nord Ovest (CEAVNO) Regione Toscana – Protocol No. 31677, University Hospital Antwerp (Antwerp, Belgium) ethics committee – Protocol No. B300201316249 and Onze Lieve Vrouw Hospital (Aalst, Belgium) ethics committee – Protocol No. B300201317801) and conducted in accordance with principles stated in the Declaration of Helsinki. Written informed consent was obtained from all patients. TMAs were constructed as previously described [25, 26]. Briefly, paraffin‐embedded tumour specimens were collected, and two pathologists selected 1 mm^2^ punches from tumour cores (with TCP above 50%) of at least 3 different tumour areas for each patient, to include in recipient tissue array blocks using a specific TMA instrument (Beecher Instruments, Micro‐Array Technologies, Silver Spring, MD). Biopsies were included in new recipient paraffin blocks and each tissue array block contained cores from at least three different tumour areas for each patient. TMA sections were de‐paraffinized with xylene and rehydrated in alcohol. Subsequently, the sections were incubated with the rabbit monoclonal antibody specific for endogenous HMGB1 (D3E5, Cell Signaling Technology), using the VENTANA BenchMark ULTRA automated slide stainer (Roche), as described previously. Negative controls were obtained by replacement of primary antibody with buffer (PBS 1X). Protein expression was determined using an Olympus BX50F bright field microscope (Olympus Optical Co Ltd., Tokyo, Japan) with a 20× objective. Scoring was performed by two blind independent observers, who also evaluated the amount of tissue loss, background staining and overall interpretability. The immunostaining intensity was classified into two grades: 0 (absent) and 1 (present). RFS was calculated from the date from first dose of chemotherapy to the date of clinical and/or radiological evidence of relapse. The Kaplan–Meier method was used to plot RFS, and the log‐rank test to compare curves. All the analyses of the samples were carried out in a blinded fashion relative to clinical outcome.

## 3 Results

### 3.1 Mass‐spectrometry‐based profiling of resected primary NSCLC tumour cohorts

Protein expression profiles of 2 independent, multi‐centre patient cohorts with NSCLC were profiled. The first cohort (discovery cohort) consisted of tumour resection samples of 45 patients with NSCLC, including both LUAD as well as LUSC diagnoses. While the analysed samples of this cohort were treatment‐naive, all patients went on to receive platinum‐based ACT following resection. The second cohort (validation cohort) consisted of 32 independent, treatment‐naive tumour resection samples. Again, both LUAD and LUSC samples were represented. Of note, while the majority of these patients also went on to receive ACT, 10 patients were left UT for later analysis of predictive vs. prognostic impact of identified biomarker candidates (Table 1, Table S1).

In both cohorts, the majority of patients (over 80%) who received ACT had stage IIA to IIIA disease, which aligns with the established standard of care for these disease stages. Likewise, as expected, > 90% of these patients received doublet chemotherapy consisting of cisplatin or carboplatin as the platinum component, and either gemcitabine or pemetrexed as combination drug. The average age at diagnosis of both cohorts was between 60 and 65 years, and both females and males were comparably represented (42/58 discovery, 50/50 validation ACT cohort). In both ACT cohorts, the ratio of LUAD to LUSC subtypes was around 60 to 40, in agreement with the observed distribution of histological subtypes of NSCLC in the Netherlands [27]. Thus, the discovery and validation cohorts analysed in this study were not only comparable but also representative in terms of clinico‐pathological parameters (Table 1, Table S1).

**Fig. 1:**
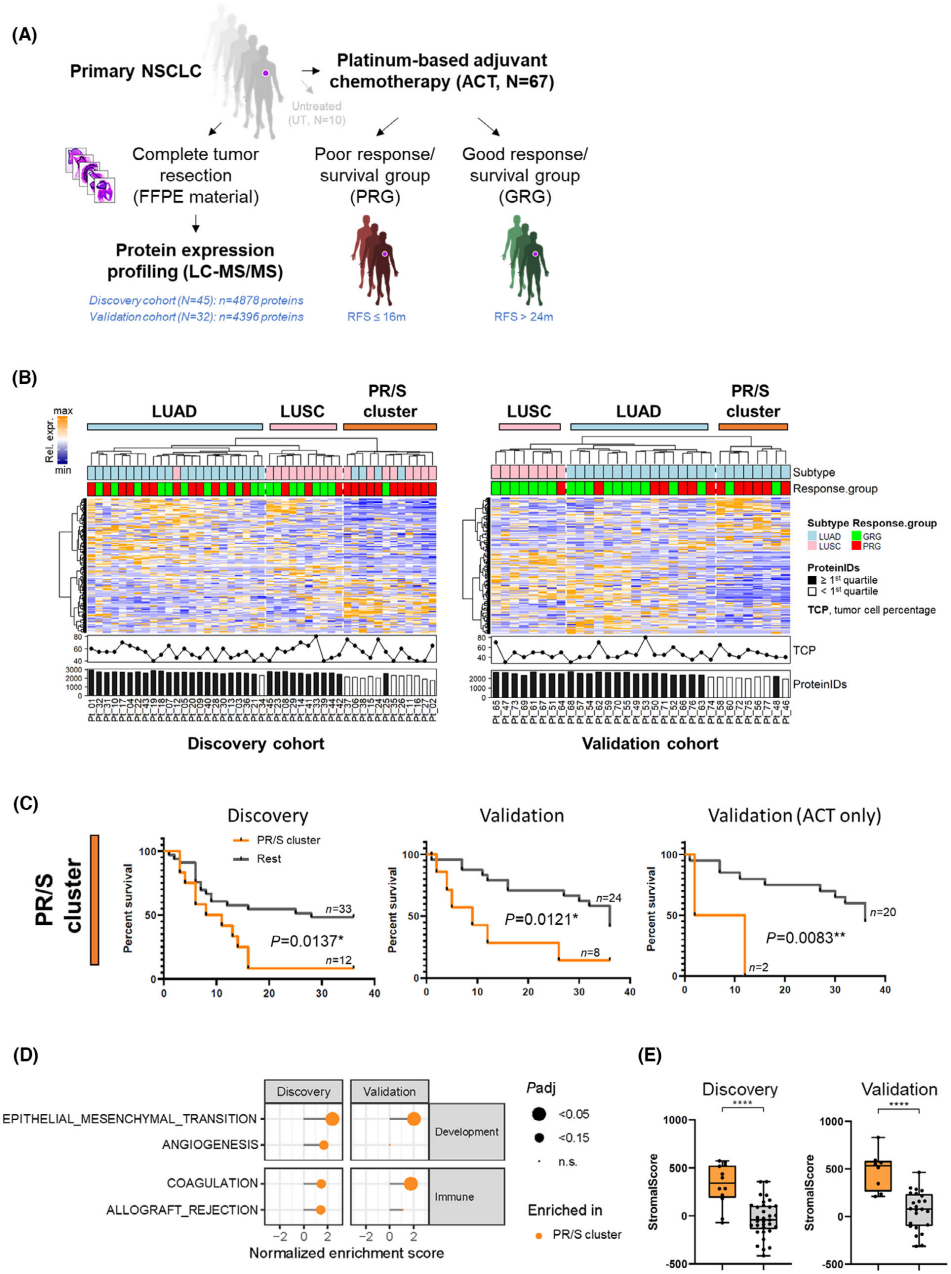
Mass‐spectrometry‐based profiling of resected primary NSCLC tumours and identification of a PR/S sub‐cluster. (A) Study design; RFS within 36 months (m) from date of first chemotherapy until recurrence (local, regional or distant) or death due to any cause. (B) Hierarchical cluster analyses based on the quantitative levels of 3561 (discovery cohort, left) or 3597 (validation cohort, right) proteins after data clean‐up and filtering (see Fig. S2A). Rel. expr., relative expression. (C) Kaplan–Meier plots of RFS of PR/S cluster vs. all other samples (Rest). *P*‐values were calculated using log‐rank test. (D) Gene‐set enrichment analysis of MSigDB Hallmark gene sets in PR/S cluster vs. all other samples. Shown are all significant (*P*adj < 0.05) and sub‐significant (*P*adj < 0.15) processes enriched in the PR/S clusters of the discovery and validation cohorts. (E) ESTIMATE analysis of PR/S cluster samples (orange) vs. all other samples (grey). **** = *P* < 0.001 (Mann Whitney test).

We performed quantitative mass‐spectrometry‐based proteomics on archived FFPE tumour resection samples of both discovery and validation cohorts. In total, we identified almost 6000 proteins (discovery: 4878, validation: 4396) in single‐shot MS‐runs, using a FDR of 1% at protein and peptide levels (Fig. 1A, Fig. S2A,B). Unsupervised hierarchical cluster analyses based on the quantitative levels of 3561 (discovery) or 3597 (validation) proteins after data clean‐up and filtering (Fig. S2A), were dominated by subtype‐specific biology, revealing distinct LUAD and LUSC clusters in both cohorts (Fig. 1B).

As a proof of principle for data quality and our statistical analysis pipeline, we performed a LUAD vs. LUSC comparison using beta‐binomial statistics to explore whether we can distinguish these histological subtypes with high sensitivity and specificity using proteins in our dataset that correspond to immunohistochemical markers used in the clinic. All of the most commonly used marker proteins, including cytokeratin 7 (KRT7), thyroid transcription factor‐1 (TTF‐1, also called NKX2‐1) and napsin‐A (NAPSA) for LUAD, as well as tumour protein p63 (TP63) and cytokeratins 5 and 6 (KRT5, KRT6) for LUSC [28, 29], were highly differential between the 2 histological subtypes in our datasets (Fig. S3). All six biomarkers were not only significantly differential (*P* < 0.05), but also had a high fold‐change (> 2.5) and, with the exception of transcription factor TTF‐1/NKX2‐1, a data presence of at least 60% in the upregulated group. This shows that relevant biomarkers can be robustly detected in these protein expression datasets, and gives confidence to the statistical methodology that we subsequently applied to identify ACT response‐related protein biomarkers. Importantly, expression characteristics (e.g. per cent data presence, abundancy and fold‐change) of all six biomarkers were very comparable between both cohorts (Fig. S3, bottom), suggesting that although different in centre of origin and sample size, expression profiling in both cohorts was reliable and representative.

### 3.2 Identification of a poor response/survival sub‐cluster (PR/S cluster)

Although dominated by subtype‐specific biology, unsupervised hierarchical cluster analyses identified 3 major sample clusters in both cohorts (Fig. 1B): a LUAD‐enriched, a LUSC‐enriched, and a third mixed subtype cluster. Strikingly, these separate sub‐clusters of mixed histology types, consisting of 12 patients (27%) and 8 patients (25%) in the discovery and validation cohorts, respectively, were significantly enriched for PR/S samples (Fig. 1C), and will thus henceforth be referred to as PR/S clusters. Furthermore, a strong correlation with low number of protein identifications was observed for these PR/S clusters, suggesting a less heterogenous protein composition which was, however, not correlated with a lower TCP (Fig. 1B). GSEA of these PR/S clusters revealed a significant enrichment of epithelial–mesenchymal transition (EMT)‐related proteins, indicative of a tumour stroma‐like subpopulation (Fig. 1D). Inference of stromal cells using the ESTIMATE algorithm indeed showed a significantly higher stromal score for PR/S cluster tumour samples (Fig. 1E). The enrichment of EMT proteins in this patient population was exemplified by the significant (*P* < 0.05) overexpression of mesenchymal proteins such as fibronectin (FN1), vimentin (VIM), nidogen 2 (NID2) and collagens (e.g. type I collagens COL1A1 and COL1A2) (Fig. S4A). Other well established extracellular matrix (ECM) proteins such as type IV collagens (eg. COL4A1), glycoproteins (eg. dermatopontin, DPT) and proteoglycans (eg. heparan sulfate proteoglycan 2, HSPG2) were likewise strongly (*P* < 0.01) overexpressed in the PR/S cluster (Fig. S4C). Of note, one quarter of all common differentially expressed proteins (DEPs) upregulated in the PR/S clusters of both cohorts could be classified as ECM proteins, the majority of which represented ECM glycoproteins and collagens (Fig. S4B). Thus both discovery and validation cohorts featured a subset of patients that showed PR/S and that clustered apart based on their protein expression profiles, which were dominated by EMT‐ and ECM‐related biology.

### 3.3 Analysis of ACT response/survival biology

**Fig. 2:**
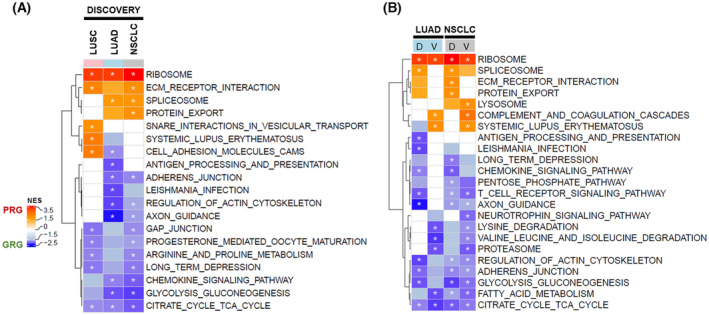
Differential biology associated with poor‐ and GRGs. (A) Heatmap showing the NESs of differentially regulated KEGG pathway signatures between PRG and GRG samples in all subtypes (LUSC and pan‐NSCLC) of the discovery cohort, as determined by GSEA. (B) Heatmap showing the NESs of differentially regulated KEGG pathway signatures between PRG and GRG samples in the LUAD and pan‐NSCLC comparisons of discovery (D) and validation (V) ACT cohorts, as determined by GSEA. Shown are the 5 most significant signatures of each comparison in each direction. Significant enrichment (*P*adj < 0.05) is annotated with *, all coloured fields without * represent sub‐significant (*P*adj < 0.15) processes, empty (white) fields are not significantly enriched (*P*adj > 0.15).

Next, we sought out to identify processes associated with proteins whose expression was specifically linked to poor and good response/survival. Based on the RFS time, RFS, patients were divided into 2 response groups: PR/S group, PRG (RFS ≤ 16 months) and good response/survival group, GRG (RFS > 24 months) (Fig. S1). We performed differential expression analysis (beta‐binomial test) comparing PRG vs. GRG samples for the LUAD and LUSC subtypes independently, as well as for the whole pan‐NSCLC cohort (LUAD and LUSC samples combined) (Tables S4 and S5). The first aim was to compare response‐related biology between the different subtypes. Due to the limited and unbalanced number of LUSC samples in the validation cohort (see Fig. S5A), this initial subtype‐specific analysis could only be performed for the discovery cohort (Fig. 2A). Gene‐set enrichment analysis revealed a strong enrichment of proteins related to the ribosome in the PRG across subtypes (normalized enrichment score, NES > 3 in all 3 comparisons). Other processes related to genetic information processing, such as spliceosome and protein export were enriched in the PRG in the LUAD and pan‐NSCLC comparisons only, while snare interactions in vesicular transport were specifically enriched in PRG LUSC. Also enriched in PRG samples across all 3 comparisons were proteins related to ECM‐receptor interaction. The most striking processes enriched in the GRG were related to carbohydrate metabolism (citrate cycle in all 3 comparisons, glycolysis in LUAD and pan‐NSCLC), immune system (chemokine signalling pathway in LUAD and pan‐NSCLC, antigen processing and presentation in LUAD only) as well as cell community and motility (adherens junction and regulation of actin cytoskeleton in LUAD and pan‐NSCLC, GAP junction in LUSC and pan‐NSCLC). As for the discovery cohort, GSEA was subsequently performed for the comparisons of the validation cohort (Fig. 2B). We focused on ACT‐response‐related biology in the LUAD and pan‐NSCLC context, since, as mentioned, the PRG vs. GRG comparison could not be performed in the LUSC validation set (underpowered PRG, see Fig. S5A). This analysis confirmed the strong enrichment of ribosome‐related biology in the PRG in both LUAD and pan‐NSCLC (NES > 3 in both validation comparisons). Likewise, the metabolic (citrate cycle, glycolysis) and actin−/adherens junction‐related processes were reproducibly significantly enriched in the GRG in the validation cohort comparisons (Fig. 2B). Other processes, such as those related to the immune system in the GRG and ECM‐receptor interaction and protein export in the PRG, could only partially be captured in the validation cohort.

### 3.4 Identification of differentially expressed proteins related to ACT response/survival

**Fig. 3:**
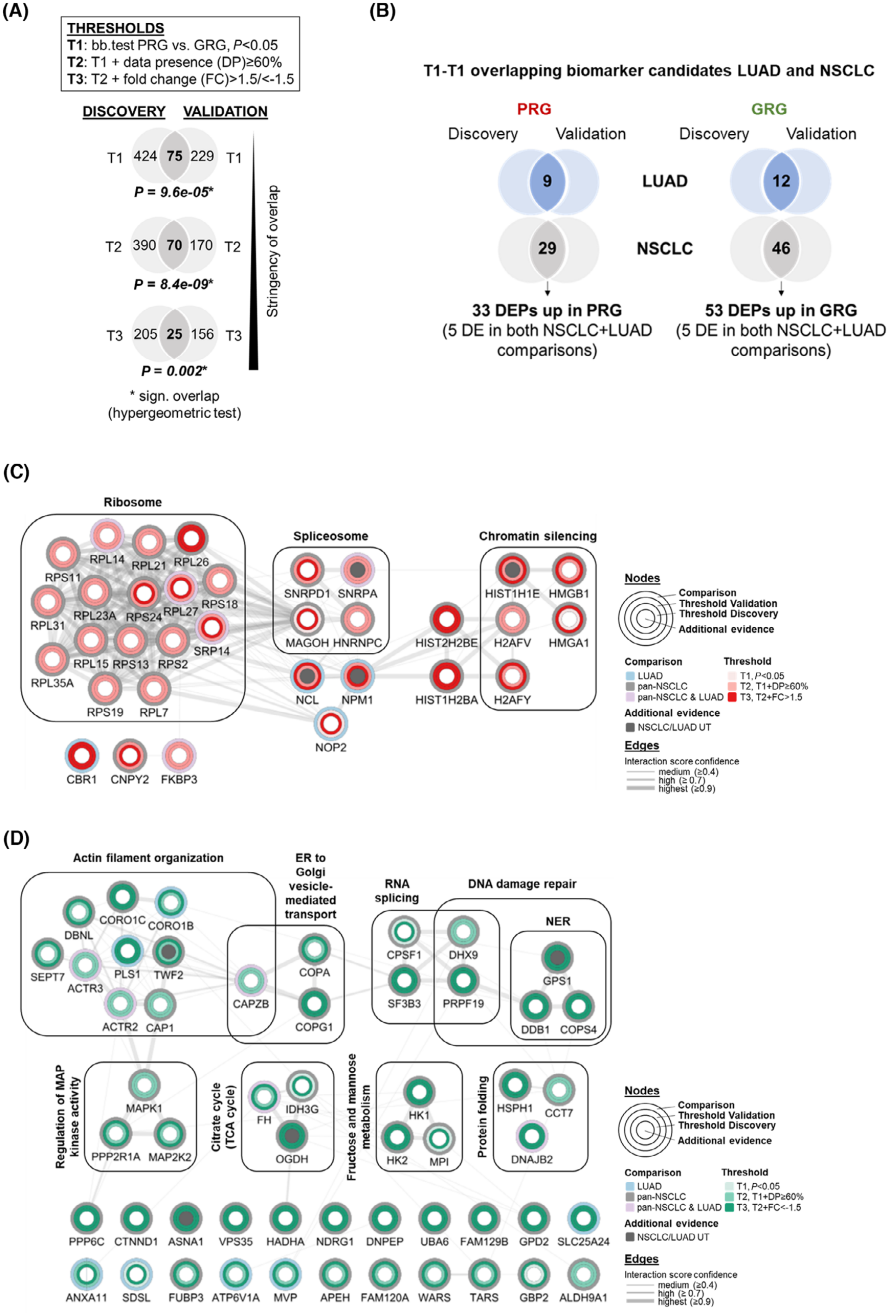
Identification of DEPs associated with poor‐ and GRGs. (A) Overlap between DEPs, as determined by beta‐binomial test, bb.test between poor and GRGs (PRG vs. GRG) in the pan‐NSCLC comparisons of the discovery and validation ACT cohorts. All significance thresholds (T1‐T3) showed a significant overlap in the number of DEPs (hypergeometric test). (B) T1‐T1 (significance threshold 1, *P* < 0.05, see Fig. 3A) overlapping biomarker candidates between discovery and validation ACT cohorts for the LUAD and pan‐NSCLC PRG vs. GRG comparisons. Due to the limited and unbalanced number of LUSC samples in the validation cohort (see Fig. S5A), this overlap analysis could not be performed for the LUSC subtype. DE(P), differentially expressed (proteins) (C) PPI network of 33 proteins with reproducibly higher abundance in PRG compared to GRG samples for the LUAD, pan‐NSCLC or both comparisons, see Fig. 3B. (D) PPI network of 53 proteins with reproducibly higher abundance in GRG compared to PRG samples for the LUAD, pan‐NSCLC or both comparisons, see Fig. 3B. The outermost node rings annotate which subtype comparison revealed differential expression, inner node rings show significance threshold for each cohort. Gray shading of inner node circle shows differential expression also in the UT subset of the validation cohort.

Encouraged by the degree of overlap on the level of enriched biology between the discovery and validation cohorts, we went on to filter for the most differential proteins between PRG and GRG and thus identify potential ACT response prediction biomarker candidates. For the discovery cohort, differential expression analysis revealed 217 significantly DEPs (p < 0.05) in the LUAD comparison, of which 83 were upregulated in PRG and 134 in GRG (Fig. S5A,B). For the LUSC subtype, this number was much larger, with 789 DEPs, of which 279 were upregulated in PRG and 510 in GRG. This discrepancy could be explained by the larger number of LUSC relative to LUAD samples in the PR/S cluster of the discovery cohort, which clustered apart based on their lower number of protein identifications and increased stromal components. Finally, the pan‐NSCLC analysis identified 550 DEPs in the discovery cohort, of which 203 were upregulated in PRG and 347 in GRG (Fig. S5A,B). In total, 259 proteins (84 PRG, 175 GRG) were differentially expressed in more than one of these subtype‐specific comparisons of the discovery cohort (Fig. S5B). Along the same lines, PRG vs. GRG comparisons were conducted for the validation cohorts. Due to the underpowered LUSC sample group in this cohort, the LUSC PRG vs. GRG comparison was excluded from all subsequent analyses. Of note, the overlap between DEPs of the discovery and validation cohort was significant, even at increasingly stringent significance thresholds (Fig. 3A). Proteins that were significantly differentially expressed (*P* < 0.05) in either or both subtype comparisons (LUAD and pan‐NSCLC) in both discovery and validation cohorts, were selected for further analysis. This concerned 86 proteins in total, 33 proteins with greater abundance in PRG samples and 53 proteins with higher expression in GRG samples (Fig. 3B).

To get a deeper functional understanding of the proteins related to poor response to ACT, the 33 PRG DEPs were visualized in a protein–protein interaction (PPI) network. Markov clustering combined with gene ontology analysis revealed 3 biologically relevant protein clusters (Fig. 3C), related to ribosome, spliceosome and chromatin silencing, respectively. Strikingly, the ribosomal protein cluster, composed of proteins of both the small (40S) and large (60S) ribosome subunits, was highly connected, with interaction scores of > 0.9 between almost all proteins. At the same time, the large majority of these proteins, while being significantly (T2 threshold; *P* < 0.05, with data‐presence in at least 60% in the upregulated group) enriched in the PRG in both discovery and validation cohorts, had relatively low fold‐change values of less than 1.5. Only RPL26 had consistent fold‐changes of just over 1.5 in both cohorts. Closely related to this cluster were other ribonucleoproteins, eg. those involved in RNA splicing (SNRPA and SNRPD1). Another cluster was made up of several DNA‐binding proteins, among which were core components of nucleosomes (eg. HIST2H2BE and H2AFY) and high mobility group proteins (HMGB1 and HMGA), both of these protein groups playing central roles in transcription regulation, DNA repair and chromatin silencing. Connecting the RNA‐ and DNA‐binding protein clusters were nucleophosmin (NPM1) and nucleolin (NCL), which have the ability to bind both chromatin and RNA. This bundled enrichment of proteins in the PRG that can directly interact with nucleic acids is intriguing. Enrichment of HMGB1 and HMGA is particularly interesting in the context of ACT response, as both high mobility families (HMGA and HMGB) display elevated affinities for the bent DNA structure of platinum‐DNA adducts [30, 31].

Analogous to the 33 biomarker candidates with elevated expression in the PRG, we had a closer look at the 53 proteins whose expression in the PRG was reduced compared to the GRG. Visualization of these GRG proteins in a PPI network (Fig. 3D) again revealed several functionally related clusters, the largest of these being actin filament organization, with proteins such as coronin‐1B and C (CORO1B and CORO1C), plastin 1 (PLS1) and actin‐related proteins 2 and 3 (ACTR2/ARP2 and ACTR3/ARP3). Several smaller clusters were enriched for proteins related to this process, such as ER to Golgi vesicle‐mediated transport (represented by eg. coatomer subunits alpha and gamma, COPA and COPG1) and regulation of MAP kinase activity (including MAP kinases MAPK1 and MAP2K2), as well as metabolic pathways (eg. citrate cycle). Most interestingly, several DNA repair proteins were also enriched in the GRG, among which DNA damage‐binding protein (DDB1), pre‐mRNA‐processing factor 19 (PRPF19) and DHX9, a BRCA1‐interacting nucleic acid helicase. Additionally, N‐myc downstream‐regulated gene‐1 (NDRG1), which has been shown to sensitize NSCLC cells to cisplatin, possibly via modulation by key NER protein ERCC1 [32], was significantly and reproducibly enriched in the GRG.

### 3.5 Differential expression of DNA damage repair‐related proteins in *in vitro* proteomic studies

Previously, we reported the proteome profiling of the conditioned media (secretomes) of a panel of NSCLC cell lines in relation to their respective cisplatin IC50 values [33]. In addition, we profiled the whole cell lysates of this NSCLC panel using LC–MS/MS. Both datasets revealed proteins associated with various biological functions implicated in (intrinsic) sensitivity to cisplatin, among which DNA repair and chromatin remodelling. Proteins involved in several relevant DNA repair pathways were significantly more abundant in cisplatin‐sensitive whole cell lysates, including PARP1, RFC1, POLD1, POLE4, UBA52, PRPF19 and DDB1 as part of NER and/or base excision repair (BER), as well as RAD50 and MSH6 as part of DSB and MMR, respectively. Additionally, DHX9, which can associate with numerous proteins involved in DNA damage response, such as BRCA1, was more abundant in cisplatin sensitive cell lines (Fig. S6A,B). Of these, we found DDB1 and DHX9 to be significantly more abundant in our clinical GRG (Fig. 3D, Fig. S6C). Interestingly, we also found DDB1 expression to be (sub‐) significantly (*P* < 0.1) higher in cisplatin‐sensitive compared to cisplatin‐induced (acquired) resistant tumour populations of small cell lung cancer (SCLC) in a proteomics study analysing genetically modified mouse models (GEMMs) [17] (Fig. S6D). Along with DDB1, other proteins involved in NER were also enriched in the cisplatin sensitive mouse lesions. In an earlier mouse study in mammary GEMMs, BRCA1‐deficient mammary tumours were found to be highly sensitive to cisplatin treatment [34]. DNA repair was among the major biological processes altered in the sensitive BRCA1‐deficient tumours after cisplatin treatment, as exemplified by induced expression of PARP1, among others. Interestingly, DHX9 expression was also found to be (sub‐) significantly (*P* < 0.1) induced upon short‐term cisplatin treatment in BRCA1‐deficient (i.e. cisplatin sensitive) but not ‐proficient mouse mammary tumours, suggesting that these proteins are part of the active response to cisplatin in responsive tumours (Fig. S6D). Taken together, these pre‐clinical data support a role for both DNA damage‐related proteins, DDB1 and DHX9, in cisplatin‐induced cell killing, thereby contributing to platinum‐drug sensitivity and favourable response, as observed in our clinical dataset.

### 3.6 Selection of top ACT response biomarker candidates

**Fig. 4:**
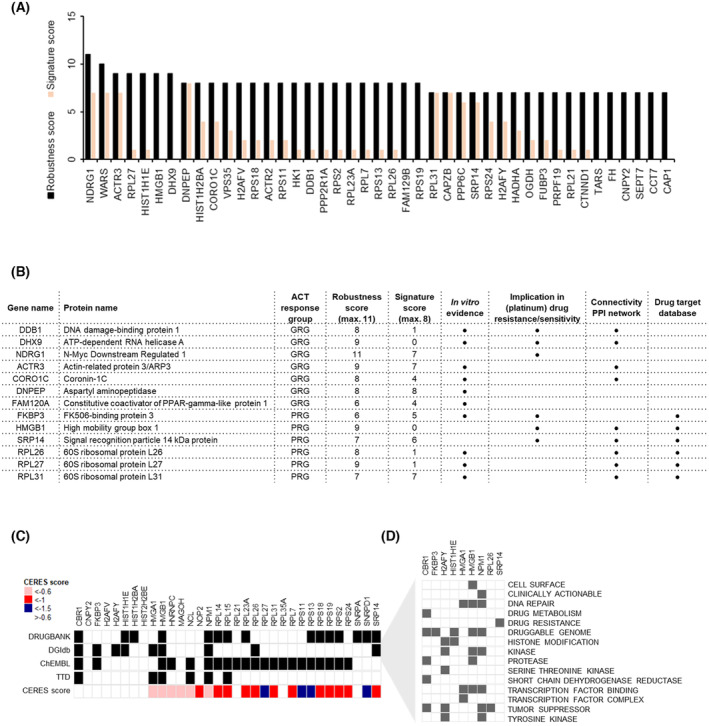
Selection of top ACT response biomarker candidates. (A) Ranking of the 43 most robust pan‐NSCLC ACT response prediction biomarker candidates (all proteins with a robustness score of 7 or higher). The cumulative robustness score was based on SC:intensity correlation, average abundance in upregulated group, significance threshold and number of unique peptides relative to the estimated molecular weight of a protein (see Table S6). The underlying signature score (orange) was based on a proteins position in the top 30 lists of best 4‐ and 5‐ protein signature combinations and whether it featured in any of the 4 stepwise logistic regression signatures (see Table S6, Fig. S7). For calculation of robustness and signature scores, see Section 2.8. (B) Table highlighting the 13 top ACT response biomarker candidates, see Fig. 3C,D; robustness and significance score, see Fig. 4A, Fig. S7C; *in vitro* evidence, see Fig. S6; drug target database, see Fig. 4C,D; implication in (platinum) drug resistance/sensitivity based on reports from literature, see main text. (C) Annotation of 33 validated PRG proteins in different drug databases. DGIdb, Drug Gene Interaction database; TTD, Therapeutic Target Database; CERES score, evaluation of genetic vulnerabilities of top PRG candidates using data from the Cancer Dependency Map Project (DepMap). Average gene essentiality scores that reflect gene dependence were calculated in 53 NSCLC cell lines, whereby a lower CERES score indicates a higher likelihood that the gene of interest is essential in a given cell line. An essential gene threshold of −0.6 CERES score was used [62]. (D) DGIdb Druggable Gene Categories.

Of the 86 consistently DEPs between GRG and PRG in both discovery and validation cohorts, 75 proteins were categorized as pan NSCLC (pan‐NSCLC comparison) ACT response prediction biomarker candidates (Fig. 3A,B). Besides significant differential expression between the two response groups, a few technical aspects were considered when aiming to identify particularly robust biomarker candidates. These included a high SC to intensity correlation as well as protein identification on the basis of more than a single unique peptide. In addition, proteins with an abundance distinctly above the detection limit were deemed to be more suitable with an eye on subsequent validation via an antibody‐based assay. Considering all these aspects, the 75 candidate proteins were assigned a robustness score (Fig. 4A, Table S6), whereby the afore highlighted proteins NDRG1 (GRG marker candidate) and HMGB1 (PRG marker candidate) were among the 7 technically most robust candidate proteins. Of note, both GRG DNA damage candidates with strong pre‐clinical evidence, DDB1 and DHX9, were also among the 25 most robust proteins (Fig. 4A). Finally, multiple predictive models were constructed using proteins with a correlation between SC and intensity > 0.75 in the combined discovery and validation dataset. Using either brute force search for all logistic models (Fig. S7A) or stepwise logistic regression starting with either the most robust protein (highest robustness score) or the protein with the highest AUC (Fig. S7B), proteins were subjected to feature selection and signature modelling and, based on this, assigned a discriminative signature score (Fig. S7C, Table S6, Section 2.8). Among the proteins with the highest signature scores were DNPEP, NDRG1, ACTR3 (GRG), RPL31, SRP14 and FKBP3 (PRG).

Based on the integration of patient tumour proteomics data with our *in vitro* cell line and mouse model proteomics data, as well as functional evidence derived from protein network and gene ontology analyses, combined with described robustness and signature scores, a list of 13 top ACT response biomarker candidates was compiled (Fig. 4B): NDRG1, DDB1, DHX9, DNPEP, ACTR3, FAM120A, CORO1C (GRG candidates), HMGB1, SRP14, FKBP3, RPL31, RPL27 and RPL26 (PRG candidates). Importantly, pre‐clinical evidence from above mentioned *in vitro* studies supported the relevance of not only DDB1 and DHX9 in this list, but also of FAM120A, CORO1C, ACTR3, DNPEP (GRG), FKBP3, RPL27 and RPL26 (PRG) (Fig. 4B). Additionally, with the exception of FAM120A, RPL31, SRP14 and FKBP3, all candidates are among the 25 technically most robust proteins (Fig. 4A).

Next, we compared PRG vs. GRG in the small set of samples from patients that were left UT (all of which were LUAD subtypes and part of the validation cohort), in order to distinguish which of the identified 86 putative ACT response predictive biomarker candidates were, in fact, more likely to be prognostic. This analysis found 8 out of the 86 candidate proteins to be differentially expressed also in the UT setting. Specifically, this concerned NCL, NPM1, SNRPA and HIST1H1E of the PRG, and TWF2, GPS1, OGDH and ASNA1 of the GRG (Fig. 3C,D). Differential expression of these proteins may therefore be indicators of RFS, irrespective of whether patients receive ACT. Importantly, none of the 13 top ACT response prediction candidates (Fig. 4B) were differentially regulated in this UT cohort, suggesting that these proteins may indeed be markers of response to ACT rather than survival.

Finally, to investigate the possibility of (co‐) targeting proteins that confer resistance to platinum‐based drugs, we consulted several drug databases: Drugbank, Drug Gene Interaction database (DGIdb), ChEMBL and Therapeutic Target Database (TTD) (Fig. 4C). All six PRG ACT response prediction candidates (HMGB1, SRP14, FKBP3, RPL31, RPL27 and RPL26) were annotated in at least one of these, with HMGB1 and FKBP3 specifically categorized as being part of the druggable genome and SRP14 annotated to be involved in drug resistance in the Drug Gene Interaction database (DGIdb, Fig. 4D). Furthermore, with the exception of FKBP3, all six PRG ACT response prediction candidates had a gene essentiality score (CERES score) of < −0.6 in a large panel of NSCLC cell lines, indicating cancer cell dependence on these genes (Fig. 4C).

### 3.7 Immunohistochemistry‐based validation of HMGB1 as a putative marker protein for poor response to ACT in NSCLC

**Fig. 5:**
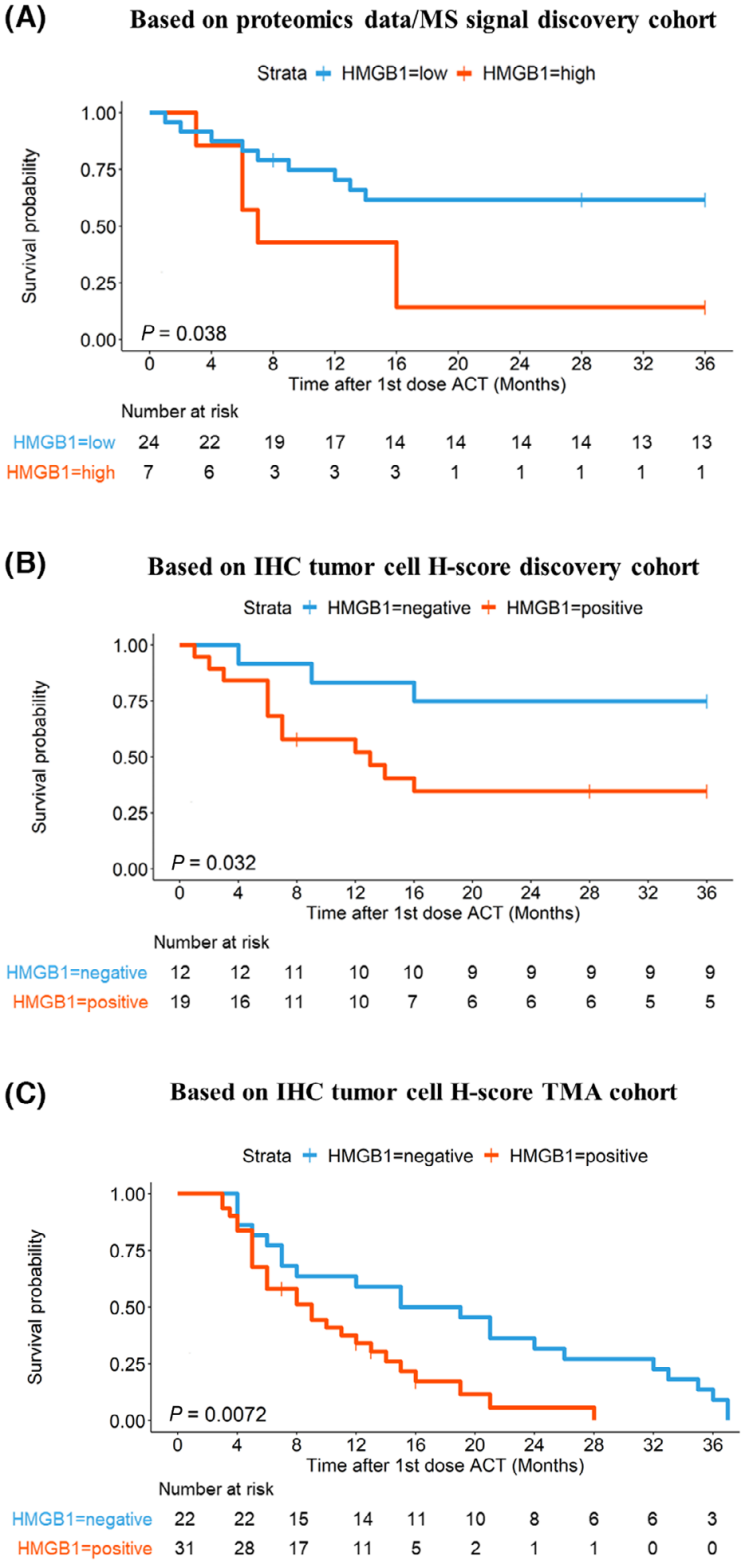
IHC‐based validation of HMGB1 as a putative biomarker for poor response to ACT in NSCLC. (A) Kaplan–Meier survival analysis (log‐rank test) of RFS based on HMGB1 protein levels in the proteomics dataset. NSCLC patient samples with the highest HMGB1 expression (top 25%, *N* = 7, red, average RFS = 12.9 months) are compared to the lower 75% (*N* = 24, blue, average RFS = 23.8 months) (B) Kaplan–Meier survival analysis of RFS based on HMGB1 staining of the same tumour sample cohort as was used for proteomic profiling. Patient samples with an IHC staining score of 0 (negative, *N* = 12, blue) vs. 10–300 (positive, *N* = 19, red) are compared. (C) Kaplan–Meier survival analysis of RFS based on HMGB1 staining of an independent TMA cohort of NSCLC patient samples (*N* = 74, of which RFS data was available for 53). Baseline demographic and clinico‐pathological characteristics of these patient samples are reported in Fig. S9. NSCLC patient samples with an IHC staining score of 0 (negative, *N* = 22, blue) vs. 10–300 (positive, *N* = 31, red) are compared. *P*‐values are based on log‐rank test.

Among the 3 most robust candidate proteins overexpressed in the PRG and part of the 13 top ACT response prediction candidates was high mobility group protein HMGB1 (Fig. 4A), suggesting this may be a particularly suitable candidate for validation using a clinically relevant antibody‐based assay. As a means of antibody‐based validation, nuclear expression of HMGB1 was initially assessed by IHC on FFPE sections of the same samples that were profiled by proteomics. To quantify HMGB1 abundance in tumour cell nuclei, H‐scores were assigned based on the proportion of cells stained and the intensity of the staining. This generated values between 0 and 300, whereby 0 was categorized as total loss and 300 as extreme gain of HMGB1 compared to normal cell staining pattern. First, correlation of mass‐spectrometry‐based HMGB1 abundance and IHC‐based HMGB1 H‐score was assessed. In a first approximation, this showed correlative tendencies but was not statistically significant (Fig. S8A,Bi). Considering the fact that the proteomics data reflected HMGB1 signal of the entire demarcated IHC slide area (which consisted of 40–80% tumour cells, but also of 20–60% tumour microenvironment including immune cells etc.), while IHC analysis specifically scored nuclear tumour cell signal, immune infiltrated tumours (with a TCP ≤ 50%) were subsequently removed from this initial correlative analysis. This resulted in a more significant correlation between proteomics‐ and IHC‐derived HMGB1 signal (Fig. S8Biii,iv). The same was true when considering only extreme gain (H‐score 300) IHC samples (Fig. S8Bii). Independent of direct signal correlation between these 2 methods (mass‐spectrometry‐based abundance and IHC‐based H‐score), Kaplan–Meier survival analysis of RFS was performed separately for data generated with either method. Strikingly, this led to very comparable results, whereby low HMGB1 abundance (proteomics) or total loss of nuclear staining (IHC) was significantly associated with longer RFS in this NSCLC ACT cohort (Fig. 5A,B). Subsequent staining and survival analysis of an independent NSCLC TMA cohort (Fig. 5C, Fig. S9) confirmed that high nuclear HMGB1 signal significantly correlates with poor disease‐related survival following ACT and may therefore have the potential to serve as a (targetable) biomarker in platinum‐based chemotherapy resistant NSCLC patient cohorts.

## 4 Discussion

Effective predictive biomarkers for the selection of patients benefiting from platinum‐based ACT in NSCLC are needed to guide treatment decisions in the clinic. This study demonstrates clear differences in the predictive value of proteomic profiles in completely resected, early stage patients with NSCLC who received post‐operative platinum‐based ACT. A reproducible sub‐cluster of around a quarter of all tumour samples was observed, that belonged to patients with poor RFS and was strongly enriched for ECM‐associated proteins. These proteins may originate from tumours that have undergone EMT, mimicking a stromal and aggressive phenotype. Alternatively, this finding could suggest that in addition to focusing on the tumour cells themselves for the mechanisms of drug resistance, the tumour stroma may play a particularly prominent role in conferring resistance to platinum‐based drugs in NSCLC tumours. Based on the analysis of the proteins identified in this sub‐population, it is tempting to speculate that the primary mechanism of protection of the tumour cells from platinum‐based ACT in this subset of samples is by providing a mechanical barrier [35, 36]. Since tumour stroma in general is characterized by higher abundancy and stiffness compared to normal ECM, combining platinum with anti‐stromal agents, such as those involved in inhibiting ECM deposition or enhancing ECM degradation, may therefore increase therapeutic effectivity in this particular patient group.

Furthermore, this study identifies candidate biomarkers for differential chemotherapy response that may allow for the selection of responsive patients beyond this PR/S cluster. We have compiled a selection of 13 such candidate proteins that represent both the prediction of ACT sensitivity and resistance, providing valuable insights for guiding treatment decisions. Among these were several proteins directly or indirectly involved in DNA damage repair. In general, DNA damage induced by platinum drugs, which significantly distort the DNA helix, is repaired by the NER mechanism. Together with DDB2, GRG candidate DDB1 is part of the initial damage recognition complex during NER. Interestingly, overexpression of DDB2 has been shown to enhance the sensitivity of human ovarian cancer cells to cisplatin by functioning as a transcriptional repressor for Bcl‐2 in combination with DDB1, thereby augmenting cellular apoptosis [37]. Upon damage sensing, subsequent repair proteins are then recruited to damaged DNA to unwind the DNA strands and excise the damaged DNA. In complex with ERCC4/XPF, ERCC1 is the rate‐limiting component of the latter process, and high ERCC1 expression is generally associated with resistance to platinum [13]. In a recent NSCLC study, ERCC1 was shown to promote resistance to chemotherapy in lung cancer cell lines, and N‐myc downstream‐regulated gene‐1 (NDRG1) was identified as an additional factor in the coordination of apoptosis and DNA damage response in hypoxic settings. Specifically, ERCC1 was shown to confer resistance to sodium glycididazole (CMNa)‐sensitized cisplatin chemotherapy by downregulating NDRG1, resulting in endurance to hypoxia and thwarted apoptosis [32]. Another study showed NDRG1 to be necessary for p53‐dependent apoptosis [38]. While the ERCC1 protein itself could not be detected by mass‐spectrometry in either discovery or validation cohorts, NDRG1 was significantly and recurrently less abundant in the PRG (i.e. more abundant in the GRG). This suggests that low NDRG1 levels could be an indirect read‐out of high ERCC1 abundance, predictive of DNA damage response and apoptosis inhibition, leading to chemotherapy resistance in NSCLC. Indeed, several other studies support the notion that downregulation of NDRG1 may be a common mechanism conferring resistance to platinum‐based chemotherapy [32, 39, 40], with Liu and colleagues not only demonstrating in a proteomics study a marked reduction of NDRG1 expression in cisplatin resistant NSCLC cells, but also showing that this downregulation was associated with the acquisition of an EMT phenotype. Other proteins among the most promising ACT response prediction candidates more directly involved in DNA repair were GRG candidate DHX9, as well as PRG candidate HMGB1. Nuclear HMGB1 has been reported to be involved in nucleotide and base excision repair (NER/BER) pathways and has been associated with chemotherapy resistance in multiple cancer types, including lung cancer [41, 42, 43, 44]. Additional studies demonstrated that downregulation or inhibition of cytoplasmic translocation was able to reverse cisplatin resistance [45, 46, 47]. Furthermore, HMGB1 is a druggable target for small molecules, such as ethyl pyruvate. Of note, this is also true for other top PRG candidates. Peptidyl‐prolyl cis‐trans isomerase (FKBP3) is a FK506 binding protein that also has a high affinity for rapamycin. It has been shown to mediate oxaliplatin resistance in colorectal cancer [48], and up‐regulation was shown to be closely correlated with poor survival in patients with NSCLC [49]. Several studies have reported a significant synergistic anti‐proliferative effect on cancer cells upon simultaneous exposure to cisplatin and mTOR inhibitors such as rapamycin [50, 51, 52, 53]. A phase I clinical trial in patients with NSCLC showed combination therapy with sirolimus (also known as rapamycin), radiation, and cisplatin to be well tolerated in these patients. As signalling through mammalian target of rapamycin (mTOR) promotes ribosome biogenesis to ensure rapid growth, the ACT resistant patient population in this study, which is characterized not only by increased expression of rapamycin binding protein FKBP3 but also a large cluster of ribosomal proteins, may be sensitive to (co‐) treatment with mTOR inhibitors. Lastly, SRP14 (signal recognition particle 14 kDa protein) is a central player in SRP‐dependent co‐translational protein targeting to the membrane (and strongly connected in the ribosomal protein cluster). Targeting of SRP14 with TAS‐103 in combination with cisplatin treatment has been shown to have a super‐additive cytotoxic effect on human small cell lung cancer cells [54, 55].

Translatability to a clinically applicable antibody‐based protein assay as well as external validation was demonstrated for one of these top ACT response biomarker candidates, HMGB1. Based on antibody availability and staining success, a next step in determining the potential of the other top ACT response prediction candidates as clinical biomarkers will require similar external validation in larger patient cohorts. Furthermore, while an UT cohort was included in this study to distinguish between predictive and prognostic potential of the candidate biomarkers, this patient population was too small to establish statistically sound conclusions in this matter.

An advantage of omics profiling in predictive biomarker research is the possibility to assess multigene molecular signatures in addition to individual biomarker candidates. We have combined these approaches by using multiple predictive signature models to help prioritize the most predictive protein biomarkers. Published predictive signatures related to prediction of ACT efficacy are almost exclusively based on mRNA expression data [56, 57, 58, 59, 60]. Proteomics‐based approaches such as those presented here could increase the functional relevance and hence future clinical application of these signatures, as protein expression is more closely aligned with cellular function and activity.

## 5 Conclusions

In this study we identify and validate key protein determinants of platinum response in patients with NSCLC, providing insights into predictive biomarkers, the landscape of platinum sensitivity and resistance mechanisms, and approaches to improve therapeutic efficacy. We demonstrate the potential of proteomic analyses on patient‐derived tumour material in constructing a biologically relevant platinum response prediction signature with clinical value. While larger studies are needed to substantiate and further validate these promising first results, clinical application of our findings holds the promise of maximizing platinum‐drug efficacy, minimizing toxicities and guiding treatment decisions towards alternative, non‐platinum‐based regimens in the future. With the advent of immunotherapy in the adjuvant therapy setting, follow‐up studies should additionally focus on predicting the sensitivity of NSCLC tumours to platinum‐based chemotherapy in the context of immunotherapy in order to enable forward‐looking personalized treatment selection.

## Conflict of interest

The authors declare no conflict of interest.

## Author contributions

CRJ, SAB and EFS conceived and designed the study; FB and SRP performed the proteomics experiments; FB performed patient selection with assistance from SAB, IB, A‐MCD and EFS; TR, KM, LMH, EG and MS were involved in acquiring and assessing clinical samples; EG coordinated the TMA analyses; TR and MS performed IHC assessment; TVP and FB performed bioinformatic analyses; FB analysed the data and wrote the manuscript; CRJ was responsible for funding acquisition. All authors read and approved the final manuscript.

## Supporting information


**Fig. S1.** Clinico‐pathological characteristics for GRG and PRG patient groups.
**Fig. S2.** Mass‐spectrometry‐based protein expression profiling of resected primary NSCLC tumours.
**Fig. S3.** Detection of clinical biomarkers used to distinguish LUAD from LUSC.
**Fig. S4.** Characterization of PR/S clusters as stromal clusters.
**Fig. S5.** Discovery of subtype‐specific response related proteins.
**Fig. S6.** Differential expression of DNA damage repair related proteins in *in vitro* proteomic studies.
**Fig. S7.** Generation of ACT response prediction signatures.
**Fig. S8.** Immunohistochemistry‐based validation of HMGB1 as a putative marker protein for poor response to ACT in NSCLC.
**Fig. S9.** Clinico‐pathological characteristics of TMA patient cohort.

**Table S1.** Clinico‐pathological characteristics of discovery and validation patient cohorts analysed by proteomics.
**Table S2.** Protein expression data discovery cohort.
**Table S3.** Protein expression data validation cohort.
**Table S4.** Beta‐binomial statistics and data annotations discovery cohort.
**Table S5.** Beta‐binomial statistics and data annotations validation cohort.
**Table S6.** Robustness and signature scores of 75 reproducibly differentially expressed pan‐NSCLC proteins.

## Data Availability

The mass spectrometry proteomics data have been deposited to the ProteomeXchange Consortium via the PRIDE [61] partner repository with the dataset identifier PXD043078. All other data supporting the findings of this study are available within the article and its supplementary information files or via the corresponding author upon reasonable request.
