# Supplementary material for: Identification of protein biomarkers for prediction of response to platinum‐based treatment regimens in patients with non‐small cell lung cancer
Source: Mol Oncol. 2024 Jan 25;18(6):1417–36. doi: 10.1002/1878-0261.13555 (PMC11161729; doi:10.1002/1878-0261.13555)
Supplement: Supplementary file 1 — Fig. S1. Clinico‐pathological characteristics for GRG and PRG patient groups. Fig. S2. Mass‐spectrometry‐based protein expression profiling of resected primary NSCLC tumours. Fig. S3. Detection of clinical biomarkers used to distinguish LUAD from LUSC. Fig. S4. Characterization of PR/S clusters as stromal clusters. Fig. S5. Discovery of subtype‐specific response related proteins. Fig. S6. Differential expression of DNA damage repair related proteins in in vitro proteomic studies. Fig. S7. Generation of ACT response prediction signatures. Fig. S8. Immunohistochemistry‐based validation of HMGB1 as a putative marker protein for poor response to ACT in NSCLC. Fig. S9. Clinico‐pathological characteristics of TMA patient cohort. [file MOL2-18-1417-s001.pdf]

Supplementary Figures

Identification of protein biomarkers for prediction of response to platinum-based treatment regimens in patients with non-small cell lung cancer

Franziska Böttger[1,2], Teodora Radonic[3], Idris Bahce[4], Kim Monkhorst[5], Sander R. Piersma[1,2], Thang V. Pham[1,2], Anne-Marie C. Dingemans[6,7], Lisa M. Hillen[8], Mariacarmela Santarpia[9], Elisa Giovannetti[1,10], Egbert F. Smit[11,12], Sjaak A. Burgers[11], Connie R. Jimenez[1,2,*]

[1]Department of Medical Oncology, Cancer Center Amsterdam, Amsterdam UMC - location VUmc, 1081 HV Amsterdam, the Netherlands
[2]OncoProteomics Laboratory, Cancer Center Amsterdam, Amsterdam UMC - location VUmc, 1081 HV Amsterdam, the Netherlands
[3]Department of Pathology, Amsterdam UMC - location VUmc, 1081 HV Amsterdam, the Netherlands
[4]Department of Pulmonary Diseases, Amsterdam UMC - location VUmc, 1081 HV Amsterdam, the Netherlands
[5]Division of Pathology, The Netherlands Cancer Institute - Antoni van Leeuwenhoek Hospital, 1066 CX Amsterdam, the Netherlands
[6]Department of Pulmonary Diseases, GROW school for Oncology & Developmental Biology, Maastricht University Medical Center, 6202 AZ Maastricht, the Netherlands
[7]Department of Pulmonary Diseases, Erasmus Medical Centre, 3000 CA Rotterdam, the Netherlands
[8]Department of Pathology, Maastricht University Medical Center, 6202 AZ Maastricht, the Netherlands
[9]Department of Human Pathology "G. Barresi", Medical Oncology Unit, University of Messina, 98125 Messina, Italy
[10]Cancer Pharmacology Lab, Fondazione Pisana per la Scienza, 56126 Pisa, Italy
[11]Division of Thoracic Oncology, The Netherlands Cancer Institute - Antoni van Leeuwenhoek Hospital, 1066 CX Amsterdam, the Netherlands
[12]Department of Pulmonary Diseases, Leiden University Medical Center, 2333 ZB Leiden, the Netherlands

Supplementary Fig. S1. Clinico-pathological characteristics for GRG and PRG patient groups……..**2**

Supplementary Fig. S2. Mass-spectrometry-based protein expression profiling of resected primary NSCLC tumors………………………………………………………………………........................…..**3**

Supplementary Fig. S3. Detection of clinical biomarkers used to distinguish LUAD from LUSC…...**4**

Supplementary Fig. S4. Characterization of PR/S clusters as stromal clusters………………………..**5**

Supplementary Fig. S5. Discovery of subtype-specific response related proteins………..…...………**7**

Supplementary Fig. S6. Differential expression of DNA damage repair related proteins in *in vitro* proteomic studies……………………………………………………………………………………..**8**

Supplementary Fig. S7. Generation of ACT response prediction signatures………………………...**10**

Supplementary Fig. S8. Immunohistochemistry-based validation of HMGB1 as a putative marker protein for poor response to ACT in NSCLC...**12**

Supplementary Fig. S9. Clinico-pathological characteristics of TMA patient cohort……………….**13**

Supplementary References…………………………………………………………………………..**14**

Supplementary Figure S1

Clinico-pathological characteristics for GRG and PRG patient groups

DISCOVERY COHORT	GRG[a]		PRG[b]		
	N	%	N	%	*p-value*[c]
**N**	19		26		
**Age, y**					
≤ 60	10	52.6	10	38.5	*0.3792*
> 60	9	47.4	16	61.5	
**Sex**					
Male	12	63.2	14	53.8	*0.5593*
Female	7	36.8	12	46.2	
**Subtype**					
LUAD	11	57.9	15	57.7	*1.000*
LUSC	8	42.1	11	42.3	
**pStage[d]**					
I	3	15.8	2	7.7	*0.6467*
II	11	57.9	13	50.0	
III	5	26.3	10	38.5	
IV	0	0.0	1	3.8	
**Platinum drug**					
Cisplatin	11	57.9	20	76.9	*0.0864*
Carboplatin	5	26.3	6	23.1	
Both, sequential	3	15.8	0	0.0	
**Combination drug**					
Gemcitabine	10	52.6	15	57.7	*0.4786*
Pemetrexed	6	31.6	10	38.5	
Other	3	15.8	1	3.8	
**TCP[e], %**					
< 60	10	52.6	17	65.4	*0.5391*
≥ 60	9	47.4	9	34.6	
**RFS[f], median (95% CI), m**	36 (34.5-37.5)		6.5 (4.7-8.3)		

VALIDATION COHORT (ACT+UT)	GRG[a]		PRG[b]		
	N	%	N	%	*p-value*[c]
**N**	19		13		
**Age, y**					
≤ 60	5	26.3	7	53.8	*0.1502*
> 60	14	73.7	6	46.2	
**Sex**					
Male	9	47.4	8	61.5	*0.4905*
Female	10	52.6	5	38.5	
**Subtype**					
LUAD	12	63.2	12	92.3	*0.1006*
LUSC	7	36.8	1	7.7	
**pStage[d]**					
I	3	15.8	3	23.1	*0.9285*
II	10	52.6	6	46.2	
III	4	21.1	4	30.8	
IV	1	5.3	0	0.0	
Unknown	1	5.3	0	0.0	
**Platinum drug**					
Cisplatin	14	73.7	6	46.2	*0.1492*
Carboplatin	0	0.0	1	7.7	
Unknown	1	5.3	0	0.0	
None	4	21.1	6	46.2	
**Combination drug**					
Gemcitabine	7	36.8	1	7.7	*0.1787*
Pemetrexed	4	21.1	5	38.5	
Both/sequential	2	10.5	1	7.7	
Other/unknown	2	10.5	0	0.0	
None	4	21.1	6	46.2	
**TCP[e], %**					
< 60	16	84.2	11	84.6	*0.9999*
≥ 60	3	15.8	2	15.4	
**RFS[f], median (95% CI), m**	36 (34.4-37.6)		7 (3.8-10.2)		

[a]GRG, good response/survival group (RFS > 24 m)
[b]PRG, poor response/survival group (RFS ≤ 16 m)
[c]Freeman-Halton extension of the Fisher exact probability test
[d]Cancer staging was performed in accordance with the TNM classification (version 7) of the Union for International Cancer Control [1]
[e]TCP, tumor cell percentage, average estimations of 2 independent pathologists
[f]RFS, recurrence-free survival within 36 m, from date of first chemotherapy (ACT sub-cohort) or date of resection (UT sub-cohort) until recurrence (local, regional or distant) or death due to any cause

Supplementary Figure S2

A

	Discovery cohort	Validation cohort
Samples	45	32
Total # Protein IDs[a]	4878	4396
Total # Protein Groups	4762	4344
Total Protein IDs LAF[b]	3561	3597
Total # Protein Groups LAF	3504	3568
Average # Proteins/Sample	2640	2461
Average # Proteins LAF/Sample	2517	2416
Min./Max./1st Quartile # of Proteins	1756/3315/2392	1960/2824/2272
Min./Max./1st Quartile # of Proteins LAF	1727/2927/2351	1926/2725/2233

[a]Identified with ≥ 1 razor+unique peptide in 1 sample at peptide and protein FDR<1%
[b]Low abundance filter, LAF: >1 norm. count in >10% of samples (discovery cohort) or ≥1 raw count in >10% of samples (validation cohort)

B

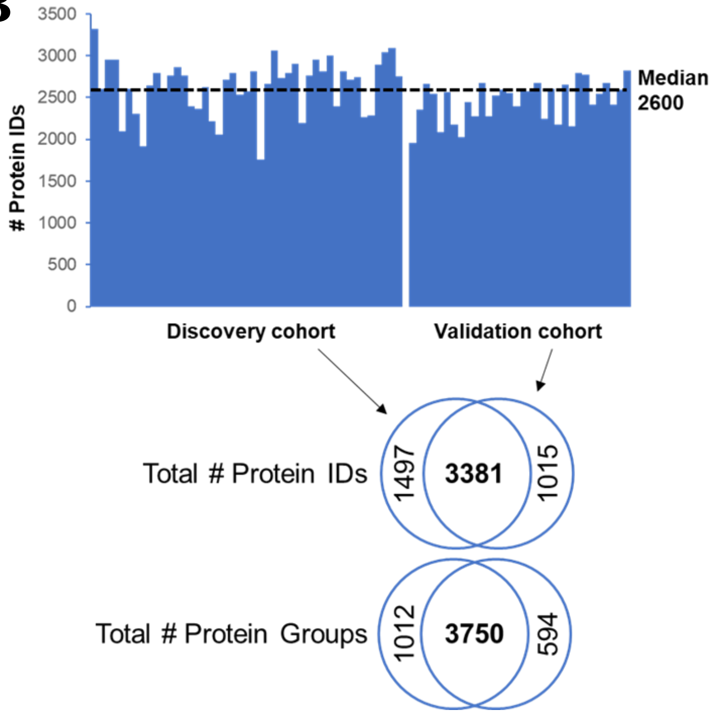

Supplementary Figure S2. Mass-spectrometry-based protein expression profiling of resected primary NSCLC tumors. A, Total number of identified protein IDs in both discovery and validation cohorts. **B,** Overlap protein IDs between discovery and validation cohorts

Supplementary Figure S3

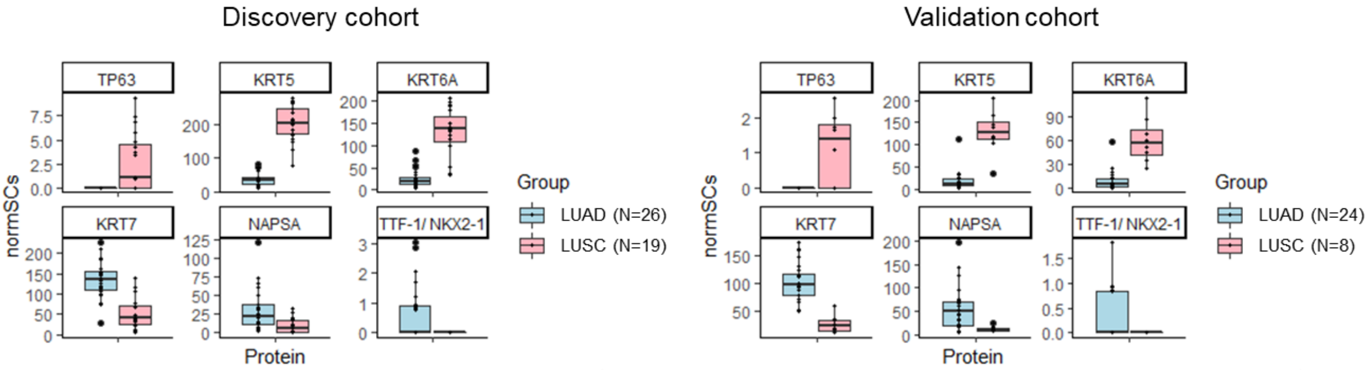

Gene name	Protein name	Marker for	p-value		Fold-change, FC		Data presence, DP (%)	
			Discovery	Validation	Discovery	Validation	Discovery	Validation
**TP63**	Tumor protein p63	LUSC	<0.00001	<0.00001	unique	unique	63.2	62.5
**KRT5**	Cytokeratin 5	LUSC	<0.00001	<0.00001	-5.7	-7.4	100	100
**KRT6A**	Cytokeratin 6	LUSC	<0.00001	<0.00001	-5.2	-7.2	100	100
**KRT7**	Cytokeratin 7	LUAD	<0.00001	<0.00001	2.5	4.0	100	100
**NAPSA**	Napsin-A	LUAD	0.0001	0.0078	3.2	4.9	100	100
**TTF-1/ NKX2-1**	Thyroid transcription factor 1	LUAD	0.0004	0.0242	unique	unique	38.5	29.2

Supplementary Figure S3. **Detection of clinical biomarkers used to distinguish LUAD from LUSC.** All 6 of the most frequently used biomarkers used to distinguish LUAD from LUSC in the clinic were significantly differentially expressed in both discovery and validation cohorts. Expression boxplots of clinical marker proteins **(top)** and differential expression statistics including p-value (beta-binomial test), fold-change and data-presence **(bottom)**

Supplementary Figure S4

A

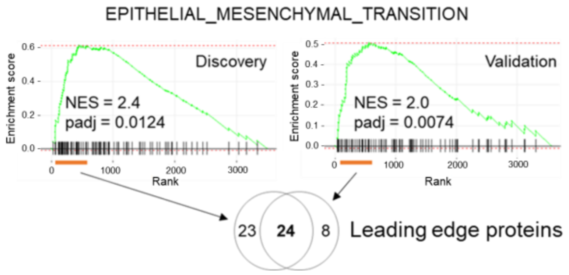

24 overlapping leading edge proteins	Discovery		Validation	
	p-value	FC	p-value	FC
COL6A3	1.12E-09	2.8	2.83E-05	2.0
COL6A2	1.77E-07	3.4	1.67E-02	1.6
COL1A2	2.02E-07	2.1	3.33E-06	2.9
COL4A1	2.20E-07	3.2	1.35E-04	2.1
NID2	5.00E-07	10.9	1.16E-02	3.4
PPIB	1.88E-05	1.4	1.36E-03	1.4
LAMC1	2.03E-05	6.1	3.58E-02	3.8
THY1	4.52E-05	2.0	6.95E-06	2.1
PCOLCE	4.60E-05	2.3	9.14E-02	2.8
FN1	5.15E-05	2.0	5.38E-03	2.3
COL4A2	1.28E-04	3.3	2.29E-03	2.2
MYL9	1.85E-04	1.6	5.06E-02	1.4
BGN	2.22E-04	1.7	2.77E-03	1.8
LUM	2.90E-04	1.7	8.21E-03	1.5
COL1A1	3.53E-04	1.8	2.88E-04	3.4
FBN1	1.47E-03	4.4	1.99E-03	7.6
POSTN	1.98E-03	3.0	2.32E-02	6.0
FBLN1	2.62E-03	2.0	2.82E-03	2.7
CD59	2.63E-03	1.4	6.31E-04	1.7
VIM	5.67E-03	1.3	4.70E-02	1.1
EFEMP2	6.67E-03	2.8	9.07E-03	3.9
THBS1	1.85E-02	1.9	2.78E-03	2.8
FMOD	2.57E-02	2.0	1.88E-05	4.1
FBLN2	7.24E-02	1.7	2.16E-03	2.4

B

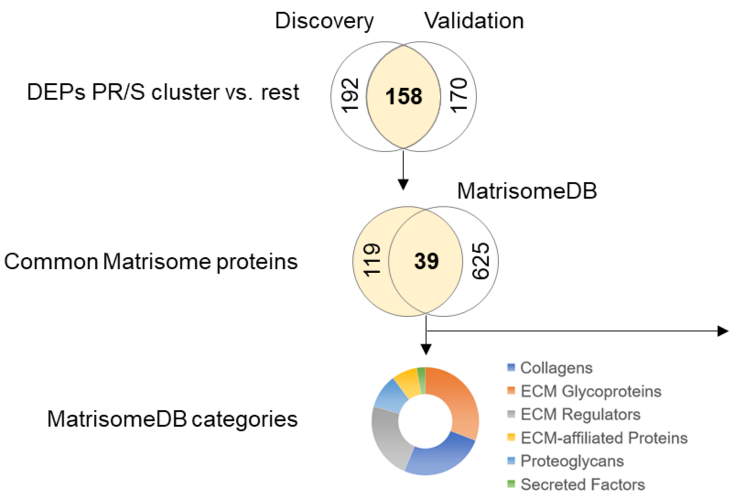

C

Common DE matrisome proteins

Proteins	Category	Discovery		Validation	
		p-value	FC	p-value	FC
COL6A1	Collagens	1.42E-06	3.6	1.21E-05	1.9
COL15A1	Collagens	3.25E-03	3.5	1.57E-03	4.6
COL6A2	Collagens	1.77E-07	3.4	1.67E-02	1.6
COL4A2	Collagens	1.28E-04	3.3	2.29E-03	2.2
COL4A1	Collagens	2.20E-07	3.2	1.35E-04	2.1
COL14A1	Collagens	1.39E-05	2.8	5.71E-03	4.7
COL6A3	Collagens	1.12E-09	2.8	2.83E-05	2.0
COL18A1	Collagens	8.15E-05	2.3	7.30E-04	1.8
COL1A2	Collagens	2.02E-07	2.1	3.33E-06	2.9
COL1A1	Collagens	3.53E-04	1.8	2.88E-04	3.4
LAMB1	ECM Glycoproteins	1.08E-07	17.2	3.04E-02	2.5
NID2	ECM Glycoproteins	5.00E-07	10.9	1.16E-02	3.4
FBN1	ECM Glycoproteins	1.47E-03	4.4	1.99E-03	7.6
EFEMP2	ECM Glycoproteins	6.67E-03	2.8	9.07E-03	3.9
DPT	ECM Glycoproteins	1.22E-08	2.4	2.47E-05	2.3
IGFBP7	ECM Glycoproteins	1.49E-02	2.2	1.18E-02	2.9
FBLN1	ECM Glycoproteins	2.56E-02	2.1	2.82E-03	2.7
FN1	ECM Glycoproteins	5.15E-05	2.0	5.38E-03	2.3
LRG1	ECM Glycoproteins	1.09E-02	2.0	7.07E-04	6.3
EFEMP1	ECM Glycoproteins	3.81E-02	2.0	8.89E-04	2.5
VWA1	ECM Glycoproteins	1.86E-02	1.9	1.03E-02	2.0
THBS1	ECM Glycoproteins	1.85E-02	1.9	2.78E-03	2.8
HRG	ECM Regulators	3.46E-02	2.0	2.46E-04	2.1
AMBP	ECM Regulators	1.53E-03	2.0	1.06E-04	2.9
CTSG	ECM Regulators	3.19E-05	2.0	2.60E-02	1.6
SERPINF1	ECM Regulators	7.83E-04	2.0	3.45E-02	1.7
ITIH4	ECM Regulators	2.14E-02	2.0	6.74E-03	2.3
CST3	ECM Regulators	2.20E-02	1.9	1.45E-04	4.1
SERPIND1	ECM Regulators	4.55E-02	1.8	2.26E-02	1.7
SERPINA3	ECM Regulators	5.19E-04	1.7	3.12E-02	2.0
SERPINC1	ECM Regulators	1.29E-02	1.6	9.33E-03	1.8
C1QB	ECM-affiliated Proteins	2.60E-04	4.8	5.72E-04	3.1
C1QC	ECM-affiliated Proteins	2.36E-04	3.4	1.26E-03	2.6
HPX	ECM-affiliated Proteins	1.67E-03	1.5	1.51E-04	1.9
HSPG2	Proteoglycans	1.89E-03	2.5	1.80E-05	3.0
FMOD	Proteoglycans	2.57E-02	2.0	1.88E-05	4.1
LUM	Proteoglycans	2.90E-04	1.7	8.21E-03	1.5
BGN	Proteoglycans	2.22E-04	1.7	2.77E-03	1.8
S100A8	Secreted Factors	6.17E-03	1.6	1.81E-02	1.7

Supplementary Figure S4. Characterization of PR/S clusters as stromal clusters. A, Common leading edge proteins of the most enriched process in the PR/S cluster (EMT, see **Fig. 1D**) and corresponding p- and fold-change (FC) values in the PR/S cluster vs. all other samples comparison. **B,** Common differentially expressed proteins (DEPs, $p<0.05$, $FC>1.5$, data-presence, $DP \geq 60\%$) in PR/S cluster vs. all other samples (rest) comparison between discovery and validation cohort (top Venn diagram), and overlap of these common proteins with MatrisomeDB [2], an ECM-protein knowledge database) (bottom Venn diagram). **C,** Overview common matrisome proteins in both discovery and validation PR/S clusters

Supplementary Figure S5

A

COHORT	Comparison	# samples PRG	# samples GRG	# Proteins	# PRG proteins T1 | T2 | T3[1]	# GRG proteins T1 | T2 | T3[1]
DISCOVERY	LUSC-ACT	11	8	3529	279 | 248 | 43	510 | 474 | 439
	LUAD-ACT	15	11	3547	83 | 62 | 37	134 | 117 | 85
	NSCLC-ACT	26	19	3561	203 | 174 | 59	347 | 309 | 189
VALIDATION	LUSC-ACT	1	7	3405	n.p.	n.p.
	LUAD-ACT	6	8	3544	122 | 96 | 75	249 | 202 | 182
	NSCLC-ACT	7	15	3597	123 | 99 | 65	225 | 155 | 127
	LUAD/NSCLC-UT	6	4	3388	113 | 85 | 75	123 | 94 | 90

→ B (Discovery rows)

[1] Thresholds: T1, bb.test PRG vs. GRG, p<0.05; T2, T1 + data presence (DP) ≥ 60%; T3, T2 + fold change (FC) >1.5/ <-1.5; n.p., not performed

B

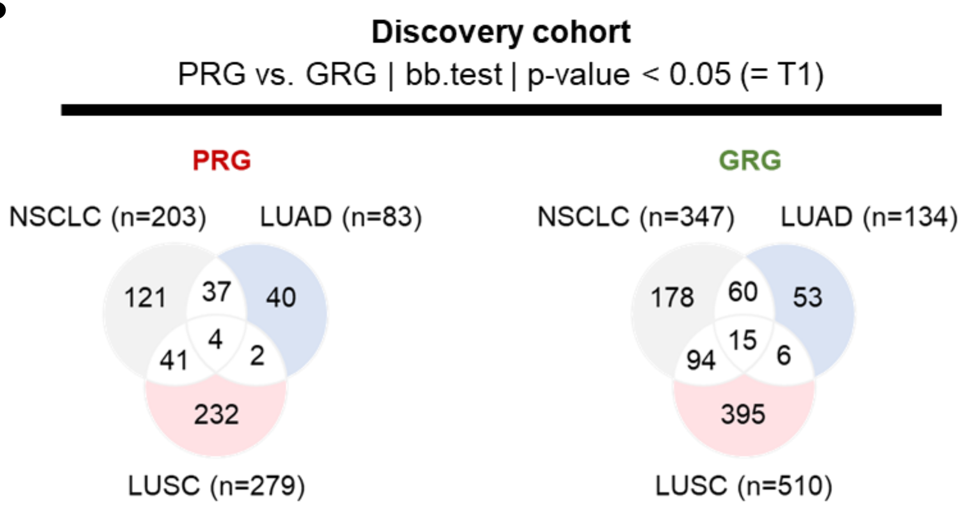

Supplementary Figure S5. Discovery of subtype-specific response related proteins. A, Overview all comparisons and number of differentially expressed proteins (DEPs). **B,** Overlap between T1-T1 (significance threshold 1, p<0.05) differentially expressed proteins between PRG and GRG in LUAD, LUSC and pan-NSCLC comparisons of discovery cohort. Due to the limited and unbalanced number of LUSC samples (see **Supplementary Figure S5A**), this overlap analysis could not be performed for the validation cohort

Supplementary Figure S6

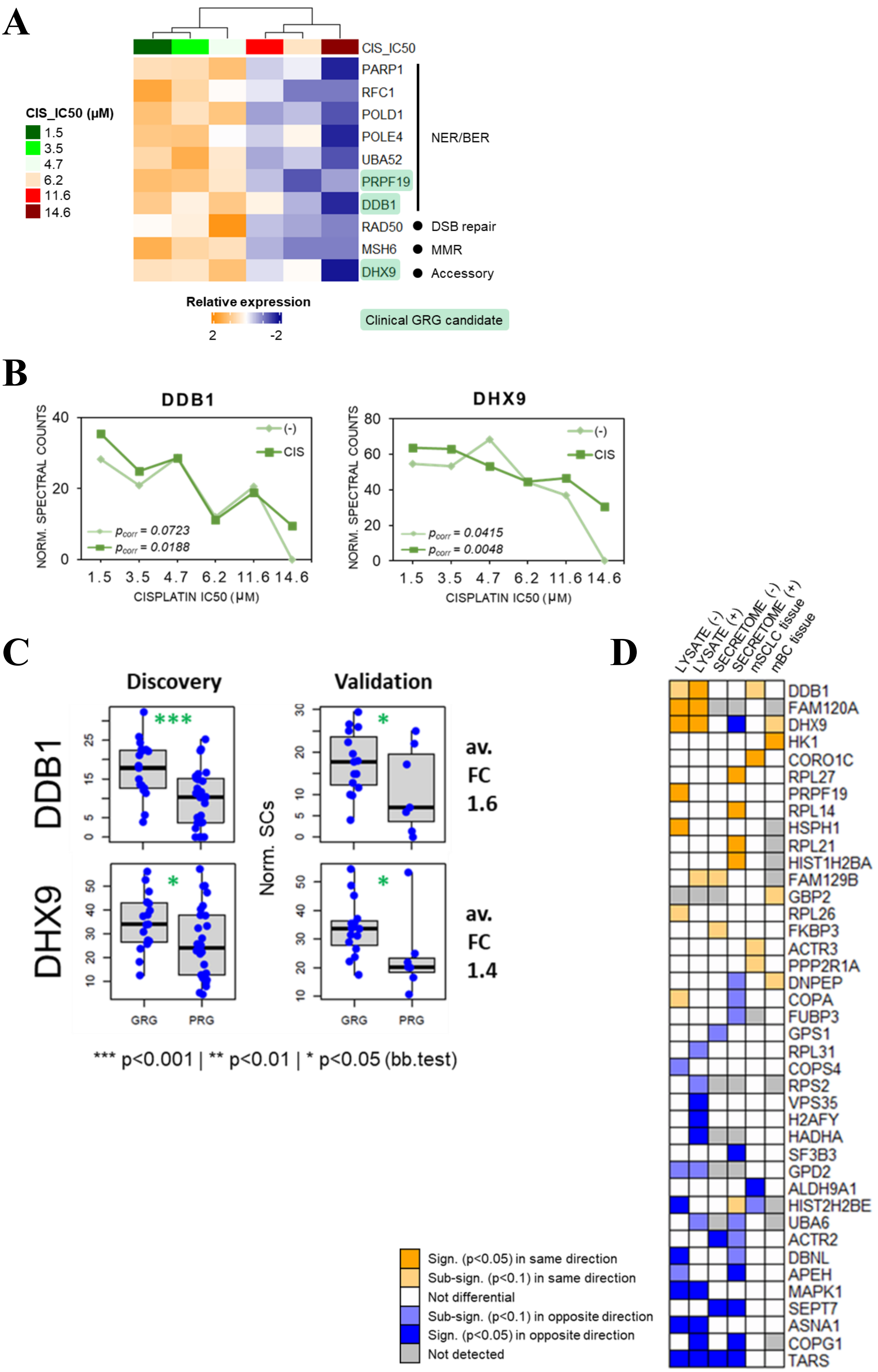

Supplementary Figure S6. Differential expression of DNA damage repair related proteins in *in vitro* proteomic studies. **A,** Relative expression of DNA damage repair related proteins in treatment-naïve whole cell lysates of NSCLC cell lines with varying sensitivities to cisplatin [4]. **B,** Expression pattern of 2 DNA damage repair related proteins in NSCLC whole cell lysates. **C,** Expression pattern of 2 DNA damage repair related proteins in clinical FFPE material of this study. **D,** Summary *in vitro* evidence for most differential, overlapping proteins related to ACT response prediction (see Fig. 3). Lysate (-), treatment-naïve whole cell lysates of NSCLC cell lines with varying sensitivities to cisplatin; Lysate (+), cisplatin-treated NSCLC whole cell lysates; Secretome (-), treatment-naïve secretomes of NSCLC cell lines with varying sensitivities to cisplatin; Secretome (+), cisplatin-treated NSCLC secretomes [4]; for both lysates and secretomes, orange colors refer to expression patterns in the same direction as in the clinical FFPE dataset of this study (i.e. up in GRG and up in cisplatin sensitive cell lines). mSCLC tissue [3], here orange colors indicate higher expression in cisplatin-sensitive compared to cisplatin-induced (acquired) resistant tumor populations of small cell lung cancer (SCLC) mouse models for GRG proteins. mBC tissue [5], here orange colors indicate induction of expression upon short-term cisplatin treatment in BRCA1-deficient (i.e. cisplatin sensitive) but not -proficient mouse mammary tumors for GRG proteins.

Supplementary Figure S7

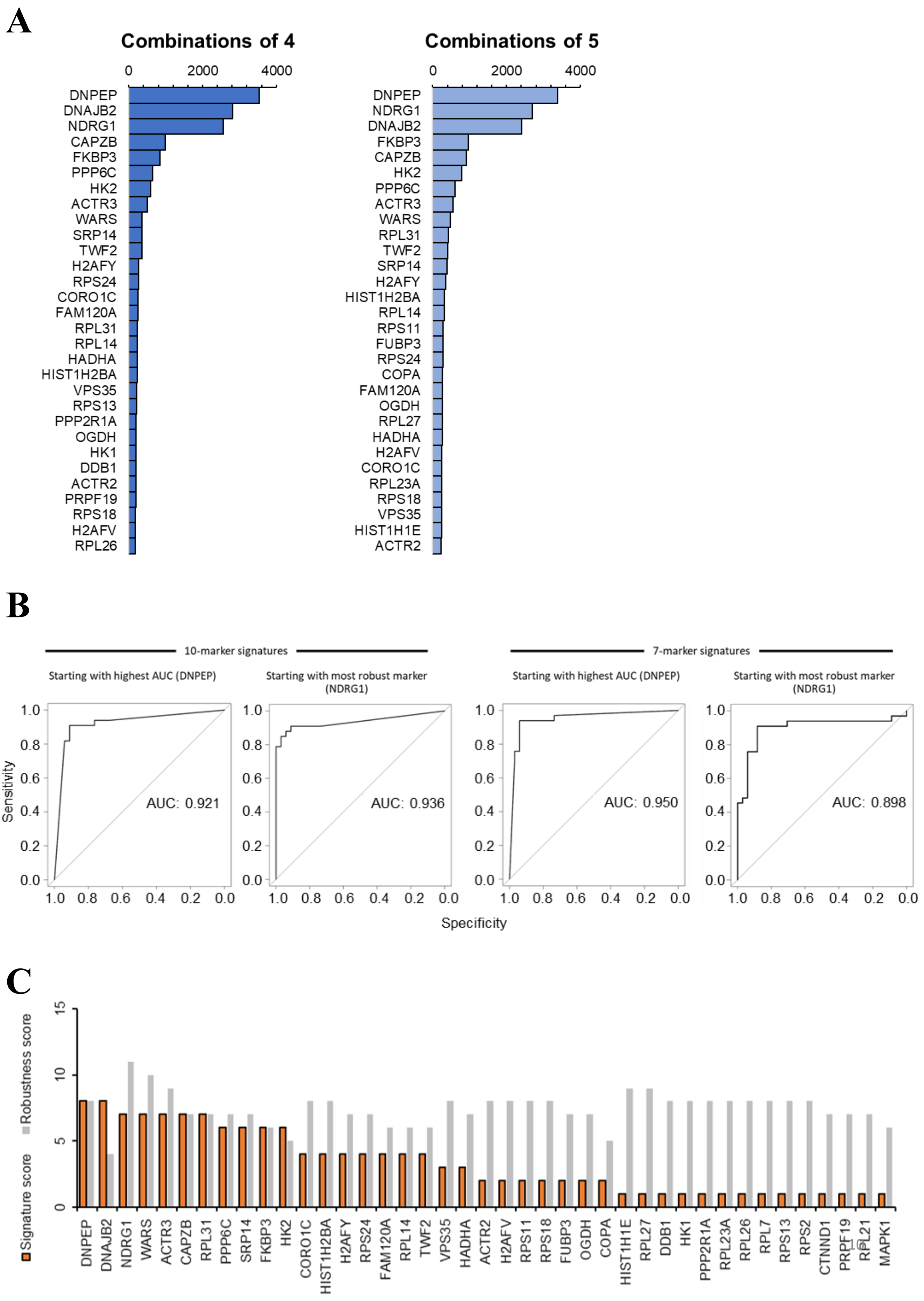

Supplementary Figure S7. Generation of ACT response prediction signatures. A, Using either brute force exhaustive search (557845 combinations, left) or random sampling (500000 combinations, right), combinations of features (different combinations of 4 or 5 proteins, respectively) were evaluated using a predictive algorithm (logistic regression). Shown are the proteins most often included in the best combinations of 4 (left) or 5 (right) proteins, with the x-axis displaying the number of appearances in the top 1% of the combinations. **B,** Stepwise logistic regression starting with either the most robust protein (NDRG1, see **Fig. 4A**) or the protein with the highest AUC (DNPEP) was performed to create 10- or 7- protein signatures. Best performing 10-marker signature featured the proteins NDRG1, DNPEP, ACTR3, RPL31, SRP14, MAPK1, RPL7, RPL21, DNAJB2 and WARS, best performing 7-marker signature the proteins DNPEP, DNAJB2, CORO1C, CTNND1, RPL31, CAPZB, RPS2 (with COPS4, SNRPD1 and GPS1 being the remaining 3 proteins completing the respective 10-marker signature starting with DNPEP). **C,** Ranking of all 41 proteins with a signature score. The signature score was based on a proteins position in the top 30 lists of best 4- and 5- protein signature combinations and whether it featured in any of the 4 stepwise logistic regression signatures (see Materials and Methods Section 2.8).

Supplementary Figure S8

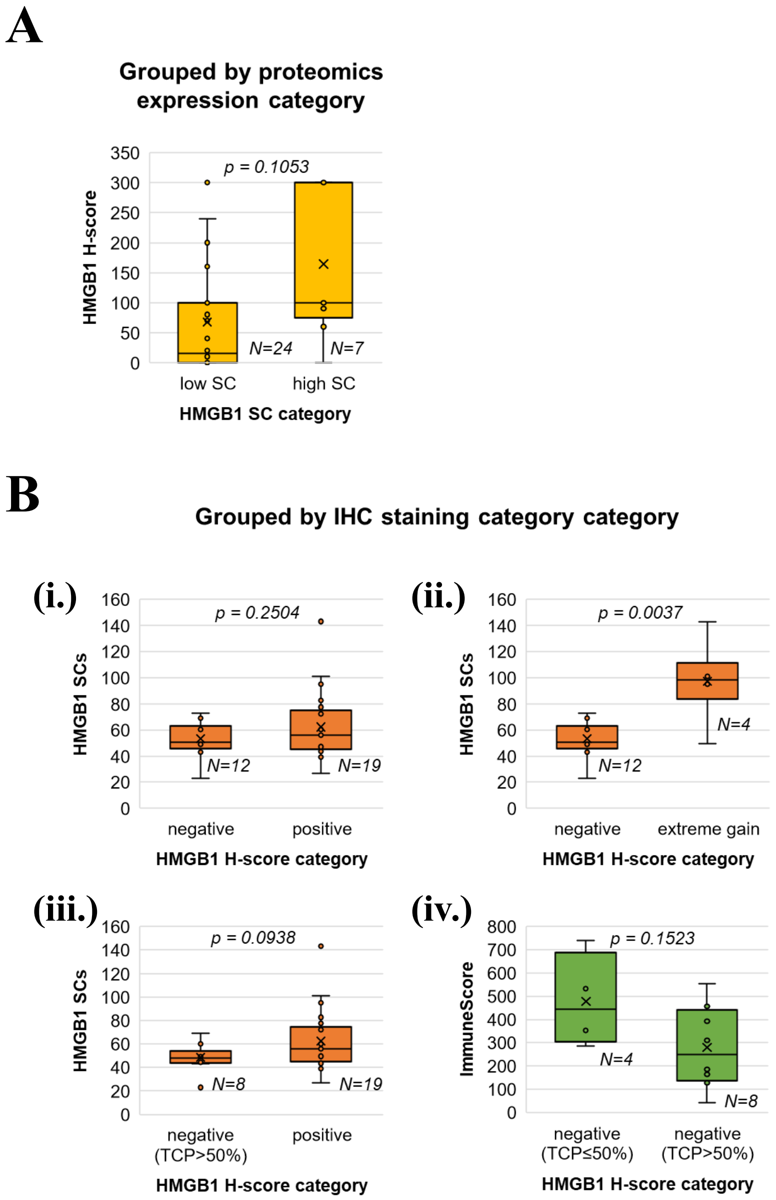

Supplementary Figure S8. Immunohistochemistry-based validation of HMGB1 as a putative marker protein for poor response to ACT in NSCLC. A, HMGB1 IHC staining scores (H-score 0-300) in NSCLC patient samples with the highest proteomics-based (SC, spectral counts) HMGB1 expression (top 25%, high SC, N = 7) are compared to the lower 75% (low SC, N = 24). **B, i.-ii.** HMGB1 protein levels based on spectral count (SC) proteomics data in NSCLC patient samples with a staining score of 0 (N = 12) versus **i.)** 10-300 (N = 19) or **ii.)** versus 300 (N=4, **ii.**) are compared **iii.-iv.** HMGB1 protein levels based on spectral count proteomics data in NSCLC patient samples with a staining score of 0 (N = 12) versus 10-300 (N = 19) are compared. HMGB1-negative tumors are split into low tumor cell percentage (TCP ≤ 50%, N=4) and high tumor cell percentage (TCP > 50%, N=8) groups (**iii.**), which correlate with higher and lower immune infiltration, respectively (ESTIMATE analysis, **iv.**). p-values are based on Student's t-test

Supplementary Figure S9

Clinico-pathological characteristics of TMA patient cohort

**Characteristic**	**No, %**
No. Patients	74
Age, years	
≤65	39 (53)
>65	35 (47)
Sex	
Male	47 (64)
Female	27 (36)
Subtype	
LUAD	48 (65)
LUSC	26 (35)
Stage	
IIA	23 (31)
IIB	30 (41)
IIIA	21 (28)
Platinum drug	
Cisplatin	57 (77)
Carboplatin	17 (23)

Supplementary References

1 Mirsadraee S. The 7th lung cancer TNM classification and staging system: Review of the changes and implications. *World J Radiol*. 2012;**4**(4).

2 Shao X, Taha IN, Clauser KR, Gao Y (Tom), Naba A. MatrisomeDB: the ECM-protein knowledge database. *Nucleic Acids Res*. 2020;**48**(D1):D1136–D1144.

3 Böttger F, Semenova EA, Song J-Y, Ferone G, van der Vliet J, Cozijnsen M, et al. Tumor Heterogeneity Underlies Differential Cisplatin Sensitivity in Mouse Models of Small-Cell Lung Cancer. *Cell Rep*. 2019;**27**(11):3345-3358.e4.

4 Böttger F, Schaaij-Visser TB, de Reus I, Piersma SR, Pham T V., Nagel R, et al. Proteome analysis of non-small cell lung cancer cell line secretomes and patient sputum reveals biofluid biomarker candidates for cisplatin response prediction. *J Proteomics*. 2019;**196**:106–119.

5 Warmoes M, Jaspers JE, Xu G, Sampadi BK, Pham T V, Knol JC, et al. Proteomics of genetically engineered mouse mammary tumors identifies fatty acid metabolism members as potential predictive markers for cisplatin resistance. *Mol Cell Proteomics*. 2013;**12**(5):1319–34.
